# RAS Pathway Inhibitors Combined with Targeted Agents Are Active in Patient-Derived Spheroids with Oncogenic KRAS Variants from Multiple Cancer Types

**DOI:** 10.1158/2767-9764.CRC-24-0582

**Published:** 2025-10-08

**Authors:** Zahra Davoudi, Thomas S. Dexheimer, Nathan P. Coussens, Thomas Silvers, Raymond G. Fox, Samantha B. Kemp, Poorva Juneja, Joel Morris, Melinda G. Hollingshead, Naoko Takebe, James H. Doroshow, Beverly A. Teicher

**Affiliations:** 1 Molecular Pharmacology Laboratory, Applied and Developmental Research Directorate, Frederick National Laboratory for Cancer Research, Frederick, Maryland.; 2 Molecular Characterization and Clinical Assay Development Laboratory, Clinical Research Directorate, Frederick National Laboratory for Cancer Research, Frederick, Maryland.; 3 Division of Cancer Treatment and Diagnosis, National Cancer Institute, National Institutes of Health, Bethesda, Maryland.

## Abstract

The Kirsten rat sarcoma viral oncogene homolog (*KRAS*) gene is among the most frequently altered genes in cancer, and the KRAS protein was long deemed undruggable. Recent strategies to target oncogenic KRAS have included both direct inhibition of the KRAS protein and indirect inhibition of its activity by targeting upstream and downstream signaling pathway mediators. A high-throughput screen of multicell-type tumor spheroids was designed to identify active combinations of targeted small molecules and KRAS pathway inhibitors. Inhibitors of the nonreceptor protein tyrosine phosphatase Src homology 2 domain–containing protein tyrosine phosphatase (SHP2) and the guanine nucleotide exchange factor Son of Sevenless homolog 1 (SOS1) were tested to evaluate indirect upstream pathway inhibition, whereas sotorasib directly inhibited the KRAS G12C variant. As single agents, sotorasib and the SHP2 inhibitor batoprotafib (TNO155) exhibited selectivity toward spheroids with KRAS G12C, whereas the SOS1 inhibitor BI-3406 showed varying activity across KRAS variants. Vertical inhibition of the rat sarcoma virus (RAS)/MEK/ERK pathway by targeting SHP2 or SOS1 and the downstream kinases MEK (trametinib) or ERK (temuterkib) was highly effective. Inhibition of upstream tyrosine receptor kinases with nintedanib in combination with batoprotafib or BI-3406 was also effective and, in combination with sotorasib, demonstrated synergy in spheroids harboring KRAS G12C. Dual inhibition of the RAS/MEK/ERK and PI3K/Ak strain transforming (AKT)/mTOR pathways by batoprotafib or sotorasib with either the mTORC1/2 inhibitor sapanisertib or the AKT inhibitor ipatasertib demonstrated combination activity, primarily in spheroids harboring KRAS G12C. The BCL-2 inhibitor venetoclax, in combination with sotorasib, batoprotafib, or BI-3406, resulted in additive and synergistic cytotoxicity. Lastly, concurrent inhibition of the KRAS pathway with sotorasib and batoprotafib demonstrated combination activity in spheroids containing KRAS G12C.

**Significance:**

KRAS variants are oncogenic drivers for a range of human cancers. Multiple combinations of small-molecule agents that target RAS signaling were screened and reduced the viability of multicell-type tumor spheroids from a variety of human solid tumors. Combinations warranting further testing were identified.

## Introduction

Rat sarcoma virus (RAS) proteins are a family of small guanosine triphosphate hydrolases (GTPases). Among them, Kirsten rat sarcoma viral oncogene homolog (*KRAS*) is a frequently altered oncogene in human cancers and is implicated in cancer types with high mortality rates, including pancreatic ductal adenocarcinoma (PDAC), non–small cell lung cancer (NSCLC), and colorectal cancer (1–4). As a GTPase, KRAS regulates signal transduction by functioning as a binary switch between its active GTP-bound and inactive GDP-bound structures. KRAS signaling is mediated by guanine nucleotide exchange factors (GEF) and GTPase-activating proteins (GAP; ref. 3). Gain-of-function variants of the residues G12, G13, and Q61 enhance GTP binding due to an increased nucleotide exchange rate and/or impaired GAP interactions and promote oncogenesis (5). KRAS mediates upstream signals from growth factor receptors such as the EGFR to downstream signaling pathways, including the MAPK signaling pathway, the PI3K/Ak strain transforming (AKT)/mTOR pathway, and the RalGEF/Ral pathway (6–8).

For decades, RAS proteins were considered “undruggable,” and RAS has been a challenging drug target due to its small size and apparent absence of allosteric binding pockets in early crystal structures. Additionally, RAS has picomolar affinities for both GDP and GTP, which poses a challenge for the development of competitive inhibitors because intracellular concentrations of GTP and GDP are in the micromolar range (9, 10). An ability to target the KRAS variant G12C followed the discovery of a novel pocket near the switch regions and proximal to the abnormal cysteine residue. This discovery enabled the development of selective inhibitors that covalently bind the reactive cysteine residue to stabilize the inactive GDP-bound structure and impair interactions with Raf (11–17). Additionally, analogs of GDP were reported that covalently bind and selectively inhibit KRAS G12C (18, 19).

The discovery of a hydrophobic switch-II groove on the surface of KRAS G12C (20) enabled the development of the lead compound MRTX1257 and clinical candidates MRTX849 (21) and AMG 510 (22). Sotorasib (AMG 510) was the first KRAS G12C inhibitor to enter a clinical trial (23), and it demonstrated activity in solid tumors with the KRAS G12C variant from heavily pretreated patients (CodeBreaK 100, clinicaltrials.gov; NCT03600883; ref. 24). Long-term clinical studies demonstrated the safety and durable efficacy of sotorasib in patients with pretreated advanced NSCLC harboring KRAS G12C (25), and sotorasib significantly increased progression-free survival compared with the standard of care, docetaxel (clinicaltrials.gov; NCT0430780; ref. 26). In 2021, the FDA granted accelerated approval to sotorasib for the treatment of locally advanced or metastatic NSCLC with KRAS G12C in adult patients (27). Sotorasib, both alone and in combination with other therapeutics, is continuing clinical trials (clinicaltrials.gov; NCT03600883, NCT05180422, NCT04303780, NCT05638295, NCT04625647, NCT05398094, and NCT05118854; refs. 6, 28).

As an alternative to direct inhibition of KRAS, several agents have advanced to clinical trials that inhibit factors upstream of RAS and thus might not be affected by RAS status. The GEF Son-of-Sevenless homolog 1 (SOS1) is required for RAS activation, and the Cdc25 domain of SOS1 binds GDP-bound RAS to catalyze the exchange of GDP for GTP (29). SOS1 also interacts with GTP-bound RAS at an allosteric site in the Ras exchanger motif domain to stabilize the SOS1 active site and augment its GEF function to promote increased catalytic activity (30, 31). Structural and functional studies of SOS1 have enabled the development of multiple SOS1 inhibitors, including BAY-293, which binds the hydrophobic pocket of the Cdc25 domain and disrupts the interaction between RAS and SOS1 (9, 32). The SOS1 inhibitor BI-3406 also binds the Cdc25 hydrophobic pocket and demonstrates improved potency and selectivity compared with previous compounds (33, 34). Exposure of cell lines expressing KRAS variants to BI-3406 led to a rapid reduction in GTP-bound KRAS and pERK levels, correlating with growth inhibition (9, 34). An analog of BI-3406, BI 1701963, is in clinical trials in patients with advanced solid tumors harboring KRAS variants, both alone and in combination with agents including irinotecan, the KRAS G12C inhibitor adagrasib, sotorasib or BI 1823911, and the MEK inhibitor trametinib or BI 3011441 (clinicaltrials.gov; NCT04111458, NCT04975256, NCT04835714, NCT04627142, NCT04973163, NCT04185883).

The initiation, propagation, and termination of biological signaling cascades are regulated by protein tyrosine kinases and protein tyrosine phosphatases (PTP). Src homology-2 (SH2) domain–containing PTP (SHP2) is a nonreceptor PTP that is recruited to activated receptor tyrosine kinases (RTK) and regulates signaling. Activation of the RAS/MEK/ERK signaling pathway by SHP2 involves the dephosphorylation of several negative regulators of RAS, including p120-RasGAP and SPRY/Sprouty, to increase GTP-bound RAS (35). Additionally, the SHP2-mediated dephosphorylation of RAS at Y32 enables binding to Raf (36). Inhibitors of the SHP2 PTP catalytic domain have been developed, and several are in clinical trials; however, the development of such inhibitors has been challenging due to limited potency, selectivity, permeability, and bioavailability (37). Novartis discovered the first allosteric SHP2 inhibitor, SHP836, from a high-throughput screen, which bound in a central tunnel at the interface of the PTP catalytic domain, the N-terminal SH2 domain, and the C-terminal SH2 domain to stabilize an auto-inhibited conformation of SHP2. Medicinal chemistry optimization of SHP836 to SHP099 resulted in a >70-fold increase in potency with improved selectivity and oral bioavailability (38, 39). Ultimately, this work enabled the discovery of the first-in-class SHP2 inhibitor, batoprotafib (TNO155), which is in clinical trials alone and in combinations, including KRAS G12C inhibitors such as sotorasib, adagrasib, or JDQ443 (clinicaltrials.gov; NCT03114319, NCT04000529, NCT04185883, NCT04330664, NCT04292119, NCT04294160, NCT04699188, NCT05490030, NCT05541159; ref. 40).

Based on the critical role of the RAS pathway in cellular function and the extensive feedback reactivation in cancers with KRAS variants, a comprehensive blockade of signal transduction is essential for anticancer activity (14, 41). Consequently, this study evaluated the KRAS G12C inhibitor sotorasib, as well as the indirect KRAS inhibitors BI-3406 and batoprotafib, both alone and in combinations with 16 FDA-approved and investigational anticancer agents. The nonclinical compounds MRTX1257, a KRAS G12C inhibitor, and BAY-293, a KRAS-SOS1 interaction inhibitor, were also tested to provide confirmatory data that did not affect the conclusions of this study and are available from the PubChem BioAssay public database. The single agents and combinations were tested in multicell-type tumor spheroids, which served as models for human solid tumors and contained malignant cells, endothelial cells, and mesenchymal stem cells (42–46). The spheroids were grown from 19 malignant cell lines, including patient-derived cell lines from the NCI Patient-Derived Models Repository (NCI PDMR) and an established NSCLC cell line, HOP-62. The malignant cell lines were derived from cancers characterized by a high frequency of *KRAS* alterations, including colorectal cancer, NSCLC, and pancreatic cancer.

## Materials and Methods

### Compounds

The drugs and investigational agents BI-3406 (NSC825286), BAY-293 (NSC824723), batoprotafib (TNO155; NSC825523), sotorasib (AMG 510; NSC818433), MRTX1257 (NSC819558), elimusertib (BAY 1895344; NSC800525), molibresib (GSK525762; NSC774829), temuterkib (LY3214996; NSC803410), venetoclax (NSC766270), alisertib (NSC759677), olaparib (NSC753686), talazoparib (NSC767125), erdafitinib (NSC781556), ipatasertib (NSC767898), cabozantinib (NSC761068), nintedanib (NSC756659), abemaciclib (NSC768073), docetaxel (NSC628503), trametinib (NSC758246), and sapanisertib (NSC764658) were obtained from the NCI Developmental Therapeutics Program Chemical Repository (47). The FDA-approved anticancer drug set is available from the Developmental Therapeutics Program. The drugs and investigational agents used in this study were demonstrated to be >95% pure by proton nuclear magnetic resonance and LC/MS. The stock solutions were prepared in DMSO (Sigma-Aldrich, cat. # D2650) at 400-fold the tested concentration and stored at −70°C prior to their use. All drugs and investigational agents were tested over a range starting from a high concentration at or near the clinical C_max_ and decreasing in half-log increments. If the clinical C_max_ for an agent had not been determined, the highest concentration tested was 10 μmol/L (Supplementary Table S1).

### Cell lines

The patient-derived cancer (PDC) cell lines 186277-243-T-J2-PDC, 253994-281-T-J1-PDC, 254851-301-R-J1-PDC, 276233-004-R-J1-PDC, 292921-168-R-J2-PDC, 323965-272-R-J2-PDC, 327498-153-R-J2-PDC, 349418-098-R-PDC, 521955-158-R2-J5-PDC, 521955-158-R6-J3-PDC, 519858-162-T-J1-PDC, 885724-159-R-J1-PDC, 931267-113-T-J1-PDC, 941728-121-R-J1-PDC, CN0375-F725-PDC, K00052-001-T-J1-PDC, K24384-001-R-PDC, and LG0567-F671-PDC (Supplementary Table S2) were obtained from the NCI PDMR. The established cell line HOP-62 was developed by Michael Alley and Michael Boyd from the NCI in collaboration with Johns Hopkins University School of Medicine (RRID: CVCL_1285; Supplementary Table S2; ref. 48). Pooled donor human umbilical vein endothelial cells (HUVEC, cat. # CC-2519) and human mesenchymal stem cells (hMSC, cat. # PT-2501) were purchased from Lonza.

### Cell culture

Similar methods were previously described from spheroid studies of different cell line panels (42–46). All cells were maintained in an incubator at 37°C and 5% CO_2_ with 95% humidity. The PDC lines were cultured according to standard operating procedures established by the NCI PDMR. Briefly, all PDCs were thawed and cultured in Matrigel-coated flasks prepared with a working solution of 1× Ham’s F-12 (F-12) nutrient mix, without supplementation (Invitrogen, cat. # 11765054), 100 U/mL penicillin-streptomycin (Invitrogen, cat. # 15140122), and 2.5% Matrigel (Corning Inc., cat. # 354248) for the first three passages. All PDCs were cultured in complete DMEM/F-12 media containing advanced DMEM/F-12 (Invitrogen, cat. # 12634028), 5% defined FBS (heat-inactivated; HyClone Laboratories Inc., cat. # SH30070.03HI), 400 ng/mL hydrocortisone (Sigma-Aldrich, cat. # H4001), 10 ng/mL human EGF recombinant protein (Invitrogen, cat. # PHG0313), 24 μg/mL adenine (Sigma-Aldrich, cat. # A2786), 100 U/mL penicillin-streptomycin (Invitrogen, cat. # 15140122), 2 mmol/L L-glutamine (Invitrogen, cat. # 25030081), and 10 μmol/L Y-27632 dihydrochloride (Tocris Bioscience, cat. # 1254). The PDCs were cultured in complete DMEM/F-12 media without 10 μmol/L Y-27632 dihydrochloride for at least two passages prior to the screen. The established cell line HOP-62 was cultured in RPMI 1640 medium, HEPES (Invitrogen, cat. # 22400105), with 10% defined FBS (HyClone Laboratories Inc., cat. # SH30070.03). The pooled donor HUVEC were cultured in endothelial cell growth medium 2 (PromoCell, cat. # C-22011), and the hMSC were cultured in mesenchymal stem cell growth medium 2 (PromoCell, cat. # C-28009). For all experiments, HUVEC and hMSC were used at passages ≤5, whereas the malignant cell lines were used at passages ≤15. Samples of all cell lines were collected at regular intervals throughout the screening process for cell authentication by short tandem repeat profiling and mycoplasma testing at Labcorp (Laboratory Corporation of America Holdings) to confirm their integrity.

### High-throughput drug combination screening

Similar methods were previously described from spheroid studies focused on different cell line panels and oncology agents (42–46). Prior to their inoculation into microplates, malignant cells, HUVEC, and hMSC were removed from flasks using TrypLE Express (Invitrogen, cat. # 12605036) and harvested by centrifugation for 5 minutes at 233 × *g*. Following the removal of the supernatant, the cells were resuspended in fresh medium and counted using a Cellometer auto T4 brightfield cell counter (Nexcelom) and trypan blue to distinguish viable cells. Multicell-type tumor spheroids were grown from a mixture of three cell types: 60% malignant cells, 25% HUVEC, and 15% hMSC, as described previously (42–46). Mixed cell suspensions of 42 μL were dispensed into the wells of 384-well black/clear round-bottom ULA spheroid microplates (Corning Inc., cat. # 3830). Cell plating densities are shown in Supplementary Table S3. Following inoculation, the microplates were transferred to an incubator (Thermo Fisher Scientific) and maintained at 37°C and 5% CO_2_ with 95% humidity. Three days after inoculation, test agents or controls were delivered to the wells of the microplates. The FDA-approved and investigational anticancer agents, prepared as 400× DMSO stock solutions, were first diluted 50-fold in media, and 6 μL were subsequently transferred to the appropriate wells of microplates containing 42 μL of cell suspension using a Tecan Freedom EVO 200 base unit (Tecan) to achieve a 1× final concentration. All anticancer agents and their combinations were tested in quadruplicate. Additionally, each microplate included a DMSO vehicle control (*n* = 16) and a cytotoxicity control (1 μmol/L staurosporine and 3 μmol/L gemcitabine, *n* = 20). After the delivery of the test agents and controls, the microplates were returned to the incubator for 7 days. Ten days after inoculation, the assay was completed with the addition of 20 μL of CellTiter-Glo 3D (Promega, cat. # G9683) to each well. Next, the microplates were placed on a microplate shaker for 5 minutes. After 25 minutes of incubation at room temperature, luminescence was measured as a surrogate indicator of cell viability using a PHERAstar FSX microplate reader (BMG Labtech).

### Analysis of viability data

Luminescence measurements from the screen were exported as comma-separated value files and imported into custom Excel spreadsheets (Microsoft) for analysis. The raw luminescence data were evaluated for quality control, filtered for outliers, and converted to a percentage of viability by normalizing to the DMSO (vehicle-treated) control. Concentration–response data were fit to the four-parameter logistic equation using the Solver Add-in in Excel.

### Analysis of combination effects

The effects of simultaneously combining agents were assessed using the Bliss independence model (47). In this model, the observed combination response is compared with a predicted combination response that is calculated from the experimentally determined response to each individual agent. Bliss independence scores nearing zero signify an additive effect, wherein the observed response closely matches the predicted outcome. A positive Bliss independence score indicates that the cell viability resulting from exposure to the combination is less than expected, demonstrating greater-than-additive cytotoxicity or synergy. A negative Bliss independence score, in which the observed cell viability is greater than expected, indicates less-than-additive cytotoxicity or antagonism.

### Volume under the viability surface

To quantify overall drug combination responses, the volume under the viability surface (VUS) was calculated using the concentration–response matrices. To enable direct comparisons between drug pairs tested at different concentration ranges, the analysis was standardized by normalizing concentration values to equal spacing between concentration levels rather than using absolute concentrations. The VUS was computed through numerical integration of viability values across all concentration points in the matrix, providing an estimate of the total response surface area. Lower VUS values indicated greater overall cytotoxicity, whereas higher values reflected weaker drug activity. All VUS values were normalized on a scale from 0 to 1, where 1 represents the highest observed viability across all combination matrices.

## Results

**Figure 1.**
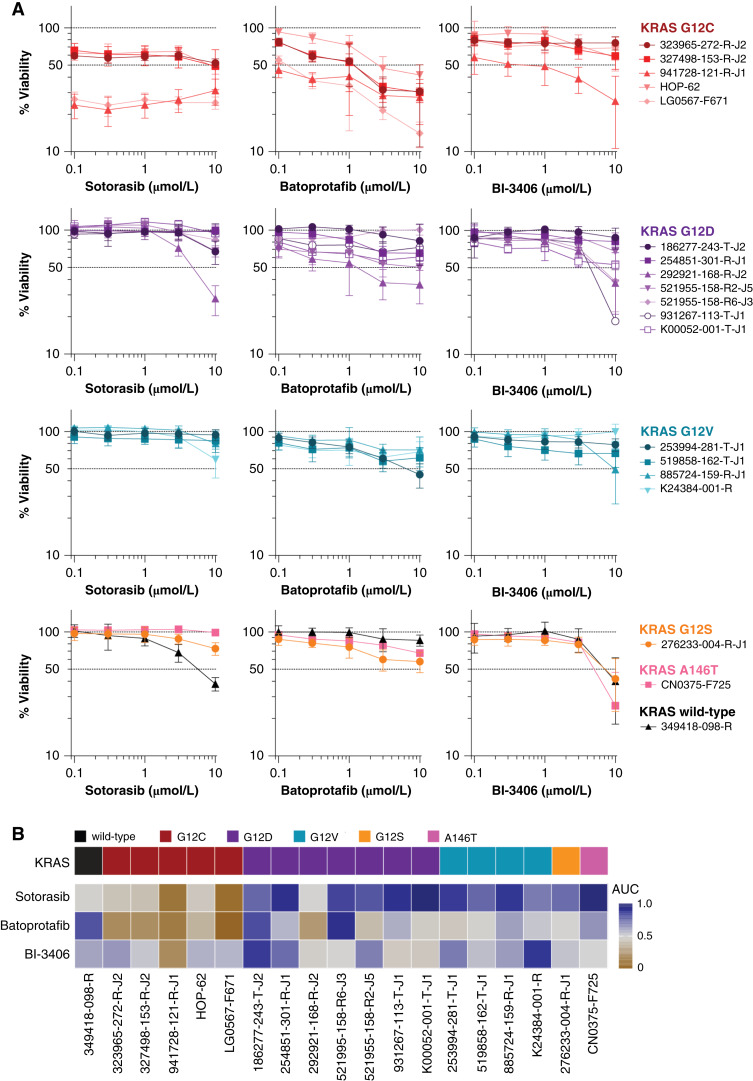
Single-agent activity of sotorasib, batoprotafib, and BI-3406 in multicell-type tumor spheroid models. **A,** Concentration–response graphs from sotorasib (left), batoprotafib (middle), and BI-3406 (right) as single agents in all 19 multicell-type tumor spheroid models. **B,** Heatmap of normalized AUC shown in **A**, with brown indicating low AUC values and blue indicating high AUC values. Annotation colors denote the *KRAS* genotype as indicated.

To mimic the tumor microenvironment and facilitate cell–cell interactions, we employed an *in vitro* multicell-type tumor spheroid model that is amenable to high-throughput screening. This model for human solid tumors comprises human malignant cells, HUVEC, and hMSC (42–46). In addition to the well-established KRAS G12C–containing HOP-62 cell line (49), 18 patient-derived malignant cell lines with a range of KRAS variants were evaluated (Supplementary Table S2). Prior to investigating drug combinations (Supplementary Table S1), the cytotoxicity of individual agents was evaluated in all the tumor spheroid models. Concentration-dependent responses from sotorasib, batoprotafib, and BI-3406 across the 19 multicell-type tumor spheroid models are shown in Fig. 1A. Sotorasib demonstrated relatively uniform responses across the concentrations chosen for the combination studies, which were selected to align with the clinical C_max_ and provide data relevant to concentrations that are achievable in patients. To assess the full sensitivity range of sotorasib, an initial experiment with nine concentrations (1 nmol/L–10 μmol/L) was conducted across all models (Supplementary Fig. S1A). Both sotorasib and batoprotafib exhibited a degree of selectivity against tumor spheroids harboring KRAS G12C, as indicated by the normalized AUC heatmap shown in Fig. 1B. Among the KRAS G12C models, some correlation between the sensitivity to sotorasib and the SHP2 inhibitor batoprotafib, as well as between the MEK inhibitor trametinib and the ERK inhibitor temuterkib, was observed based on AUC values. However, given the limited number of KRAS G12C models in this study (*n* = 5), only the correlation between trametinib and temuterkib achieved statistical significance (Pearson *r* = 0.96, *P* = 0.009). A Pearson’s correlation matrix summarizing these relationships is shown in Supplementary Fig. S1B. No correlation was observed between the doubling time of cell lines and their sensitivity to sotorasib (Supplementary Fig. S1C and S1D). In contrast, BI-3406 showed no apparent selectivity and reached an IC_50_ in approximately half of the tumor spheroids tested. All tumor spheroid models that harbor KRAS G12C demonstrated concentration-dependent responses to sotorasib at concentrations below 100 nmol/L; however, the depth of cytotoxicity varied (Supplementary Fig. S2A). The most responsive model, 941728-121-R-J1, showed a loss of heterozygosity for *KRAS* as well as a copy-number amplification (Supplementary Table S2). Although not included in this study, the pancreatic carcinoma cell line MIA PaCa-2, grown as multicell-type tumor spheroids, showed a strong concentration-dependent response to sotorasib (Supplementary Fig. S2A). No cytotoxic responses by the HUVEC and hMSC were observed from sotorasib throughout the concentration range, which likely accounted for the reduced cytotoxicity observed in multicell-type tumor spheroids compared with spheroids grown from only MIA PaCa-2 cells (Supplementary Fig. S2B). Substantially more cytotoxicity was observed from the 3D spheroids compared with monolayers, which is consistent with previous studies of KRAS G12C–targeted agents in cell lines that harbor KRAS G12C (16, 17, 22). Among a selection of seven drugs tested in combination with sotorasib, overall limited cytotoxicity was observed from the HUVEC and hMSC, except for the highest concentrations of trametinib, temuterkib, nintedanib, and venetoclax (Supplementary Fig. S3).

**Figure 2.**
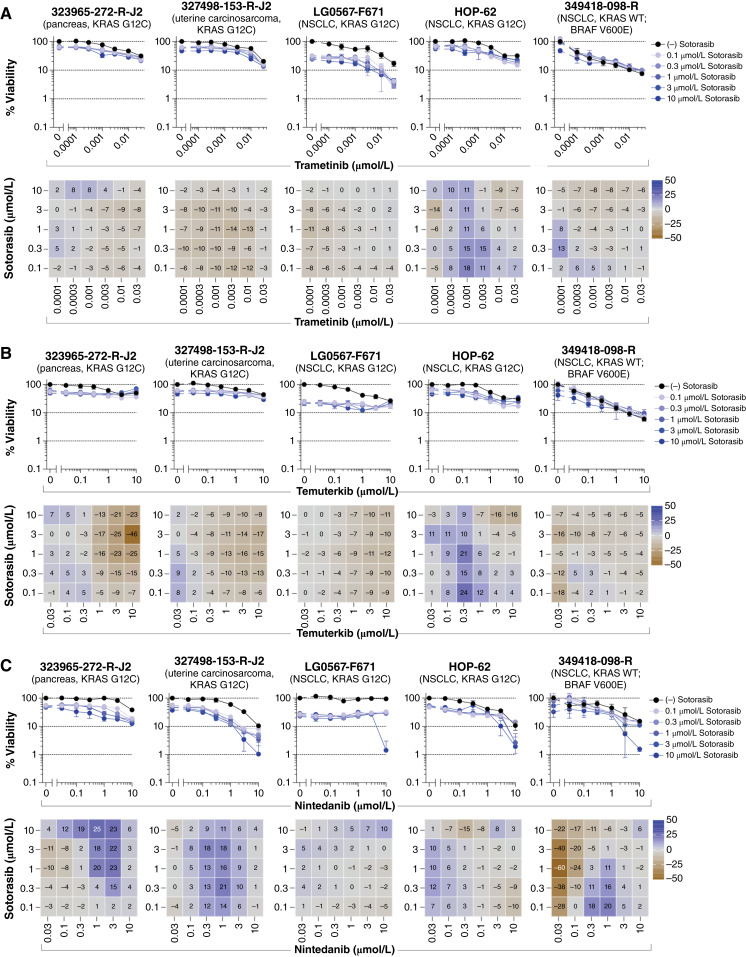
Vertical inhibition of the RAS pathway by trametinib, temuterkib, or nintedanib in combination with sotorasib. Concentration–response graphs (top, mean ± SD, *n* = 4 technical replicates) from combinations of (**A**) trametinib, (**B**) temuterkib, or (**C**) nintedanib with sotorasib are shown, with corresponding Bliss independence scores from each combination’s concentration matrix (bottom, mean of *n* = 4 technical replicates) displayed numerically and as a heatmap (blue indicates synergy, gray indicates additivity, and brown indicates antagonism). The malignant cell line name, tumor type, and KRAS status are indicated above each set of graphs. WT, wild-type.

Direct targeting of KRAS G12C with sotorasib in combination with downstream components of the RAS/MEK/ERK pathway, including trametinib or temuterkib (LY3214996), resulted in minimal additive effects or no significant combination response. Fig. 2A and B display the concentration–response graphs and Bliss independence heatmaps for four of the five tumor spheroid models containing the KRAS G12C variant, along with a NSCLC model with wild-type KRAS and a BRAF V600E variant (349418-098-R). Any observed additive effects were consistent across all concentrations of sotorasib, reflecting the relatively flat single-agent responses over the concentrations tested. Inhibition of upstream receptors in the KRAS pathway using the multitargeted RTK inhibitor nintedanib (targeting FGFR1-3, platelet-derived growth factor receptor α/β, and VEGFR1-3), in combination with sotorasib, resulted in combination effects in the tumor spheroid models harboring KRAS G12C (Fig. 2C). Notably, the pancreatic model 323965-272-R-J2 and the uterine carcinosarcoma model 327498-153-R-J2 exhibited some synergy. Unexpectedly, sotorasib demonstrated single-agent activity against the 349418-098-R model with wild-type KRAS (Figs. 1A and 2) and the 292921-168-R-J2 model with KRAS G12D (Fig. 1A), resulting in <50% cell viability at 10 μmol/L. The five models harboring KRAS G12C all demonstrated concentration-dependent reductions in cell viability from sotorasib concentrations below 100 nmol/L (Supplementary Fig. S2), whereas the 349418-098-R and 292921-168-R-J2 models demonstrated sensitivities above 1 μmol/L. Therefore, the responses observed from these two models might be attributed to off-target activity. The variant status of key signaling proteins from the NCI PDMR PDC cell lines is shown in Supplementary Table S4, and the gene copy number is indicated in Supplementary Table S5 (also see oncoprint in Supplementary Table S6).

**Figure 3.**
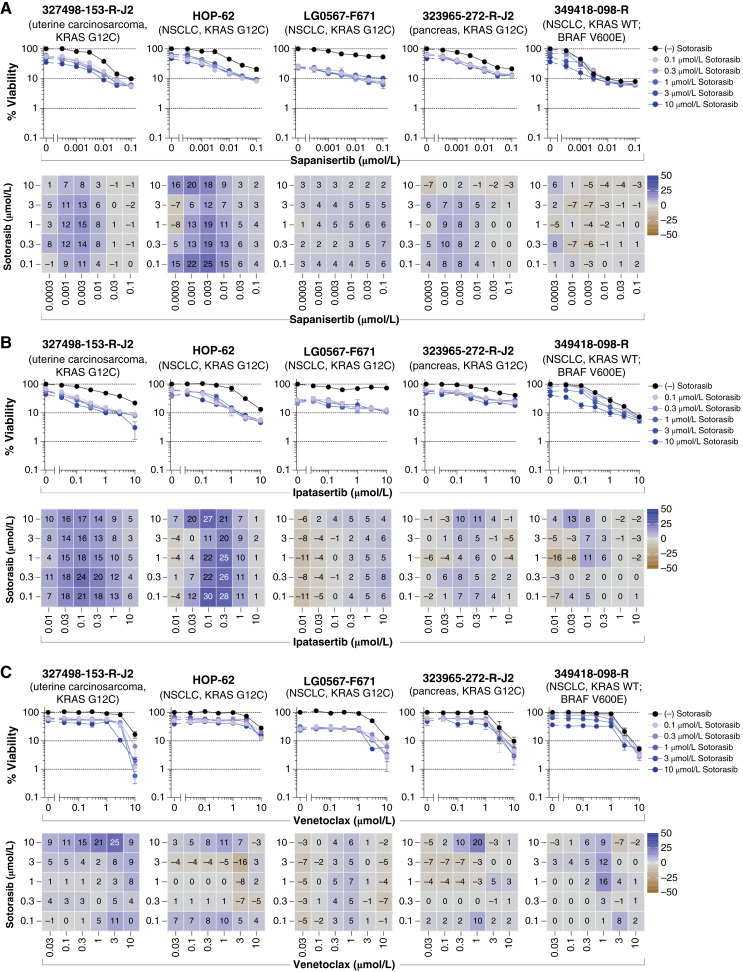
PI3K/AKT/mTOR or apoptosis pathway targeted agents in combination with sotorasib. Concentration–response graphs (top, mean ± SD, *n* = 4 technical replicates) from combinations of (**A**) sapanisertib, (**B**) ipatasertib, or (**C**) venetoclax with sotorasib are shown, with corresponding Bliss independence scores from each combination’s concentration matrix (bottom, mean of *n* = 4 technical replicates) displayed numerically and as a heatmap (blue indicates synergy, gray indicates additivity, and brown indicates antagonism). The malignant cell line name, tumor type, and KRAS status are indicated above each set of graphs. WT, wild-type.

Dual targeting of KRAS G12C and the PI3K/AKT/mTOR pathway represents another potential therapeutic strategy. The combination of sotorasib with the mTORC1/2 inhibitor sapanisertib or the AKT inhibitor ipatasertib exhibited similar additive effects in the G12C variant–containing spheroids, with the greatest synergy in the NSCLC HOP-62 model (Fig. 3A and B). Given the role of apoptotic regulation in KRAS-driven tumors, we also explored the combination of sotorasib with the BCL-2 inhibitor venetoclax. This combination primarily resulted in additive responses across spheroids harboring the KRAS G12C variant (Fig. 3C). As with previous combinations, any observed effects remained largely consistent across all sotorasib concentrations.

**Figure 4.**
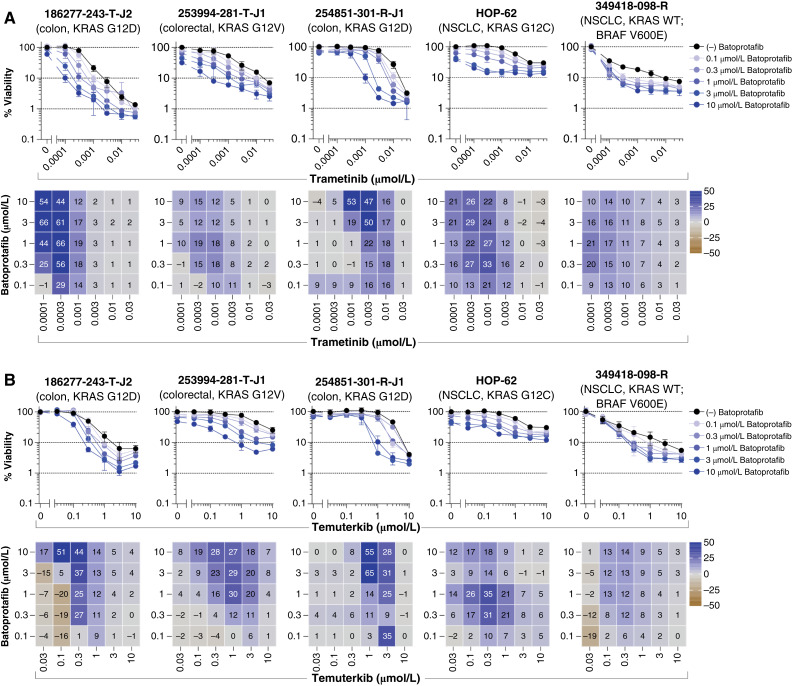
Vertical inhibition of the RAS pathway with batoprotafib in combination with trametinib or temuterkib. Concentration–response graphs (top, mean ± SD, *n* = 4 technical replicates) from combinations of batoprotafib with (**A**) trametinib or (**B**) temuterkib are shown, with corresponding Bliss independence scores from each combination’s concentration matrix (bottom, mean of *n* = 4 technical replicates) displayed numerically and as a heatmap (blue indicates synergy, gray indicates additivity, and brown indicates antagonism). The malignant cell line name, tumor type, and KRAS status are indicated above each set of graphs. WT, wild-type.

**Figure 5.**
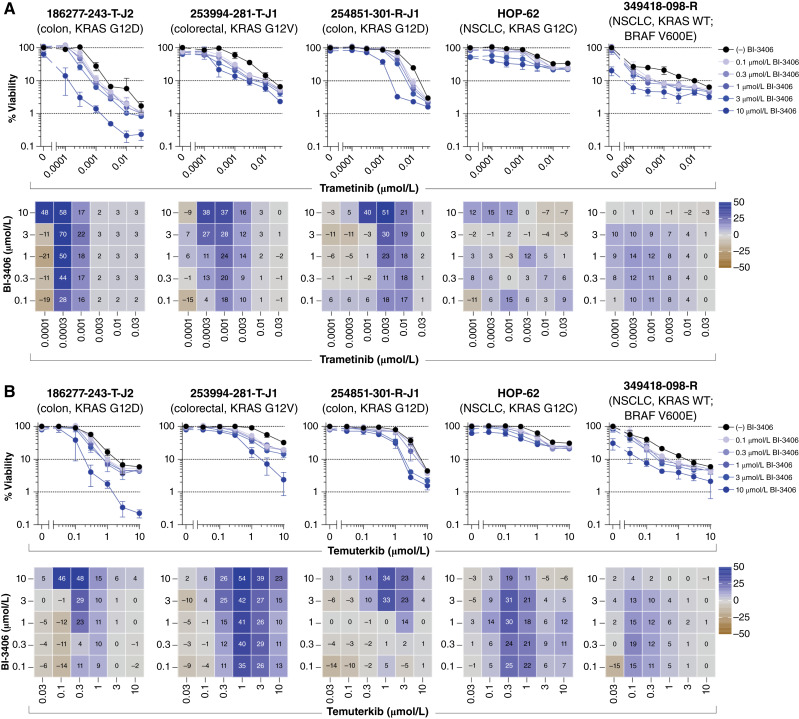
Vertical inhibition of the RAS pathway with BI-3406 in combination with trametinib or temuterkib. Concentration–response graphs (top, mean ± SD, *n* = 4 technical replicates) from combinations of BI-3406 with (**A**) trametinib or (**B**) temuterkib are shown, with corresponding Bliss independence scores from each combination’s concentration matrix (bottom, mean of *n* = 4 technical replicates) displayed numerically and as a heatmap (blue indicates synergy, gray indicates additivity, and brown indicates antagonism). The malignant cell line name, tumor type, and KRAS status are indicated above each set of graphs. WT, wild-type.

**Figure 6.**
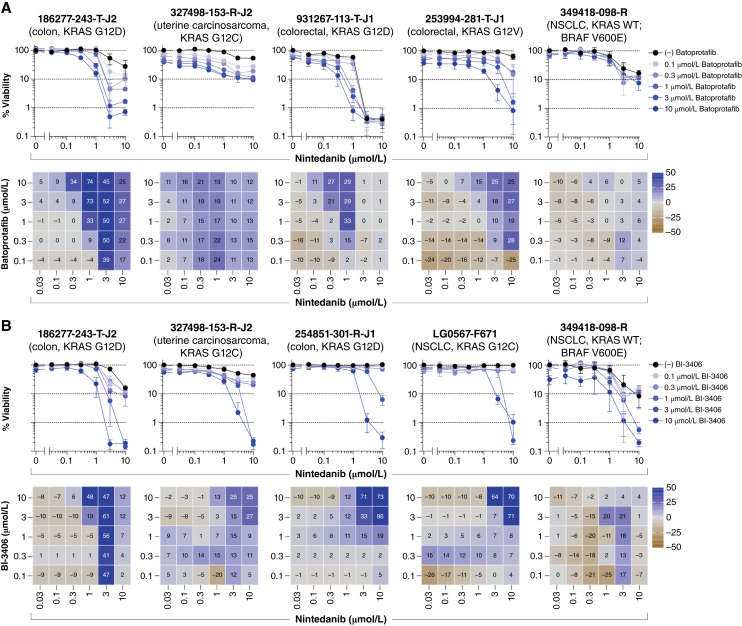
Receptor tyrosine kinase inhibition with nintedanib in combination with batoprotafib or BI-3406. Concentration–response graphs (top, mean ± SD, *n* = 4 technical replicates) from combinations of (**A**) batoprotafib or (**B**) BI-3406 with nintedanib are shown, with corresponding Bliss independence scores from each combination’s concentration matrix (bottom, mean of *n* = 4 technical replicates) displayed numerically and as a heatmap (blue indicates synergy, gray indicates additivity, and brown indicates antagonism). The malignant cell line name, tumor type, and KRAS status are indicated above each set of graphs. WT, wild-type.

Indirect targeting of KRAS with SHP2 or SOS1 inhibitors, along with downstream components of the RAS/MEK/ERK pathway, proved to be among the most effective strategies *in vitro*. This was apparent from the combination of batoprotafib with either trametinib or temuterkib. Figure 4 shows the concentration–response graphs and Bliss independence heatmaps from selected multicell-type tumor spheroid models, whereas data from all models are shown in Supplementary Figs. S4 and S5. Comparable levels of additive or synergistic effects, as indicated by the mean Bliss scores, were evident from the combinations of batoprotafib with trametinib or temuterkib across the same tumor models (Pearson *r* = 0.71, *P* = 0.0007, Supplementary Fig. S6A). Similar results were observed from BI-3406 in combination with either trametinib or temuterkib. Figure 5 shows the concentration–response graphs and Bliss independence heatmaps from selected multicell-type tumor spheroid models, whereas data from all models are shown in Supplementary Figs. S7 and S8. There was a weaker but statistically significant correlation between the mean Bliss scores from BI-3406 combinations with either trametinib or temuterkib (Pearson *r* = 0.5, *P* = 0.033; Supplementary Fig. S6B). Interestingly, statistically significant correlations were calculated between mean Bliss scores from the combinations of batoprotafib or BI-3406 with trametinib (Pearson *r* = 0.74, *P* = 0.0004; Supplementary Fig. S6C) or temuterkib (Pearson *r* = 0.85, *P* < 0.0001; Supplementary Fig. S6D), potentially attributable to the shared point at which batoprotafib and BI-3406 interfere with the RAS signaling pathway. Alternatively, upstream RTK inhibition by nintedanib also resulted in both additive and synergistic effects with batoprotafib and BI-3406 in multiple tumor spheroid models. These effects were evident at the higher concentrations of each combined drug. Figure 6 shows the concentration-response graphs and Bliss independence heatmaps from selected multicell-type tumor spheroid models, whereas data from all models are shown in Supplementary Figs. S9 and S10. Notably, combinations of nintedanib with either batoprotafib or BI-3406 were effective against cell lines with a range of KRAS variants. Similar to combinations with trametinib or temuterkib, they displayed limited activity against the NSCLC model with wild-type KRAS and a BRAF V600E variant (349418-098-R).

**Figure 7.**
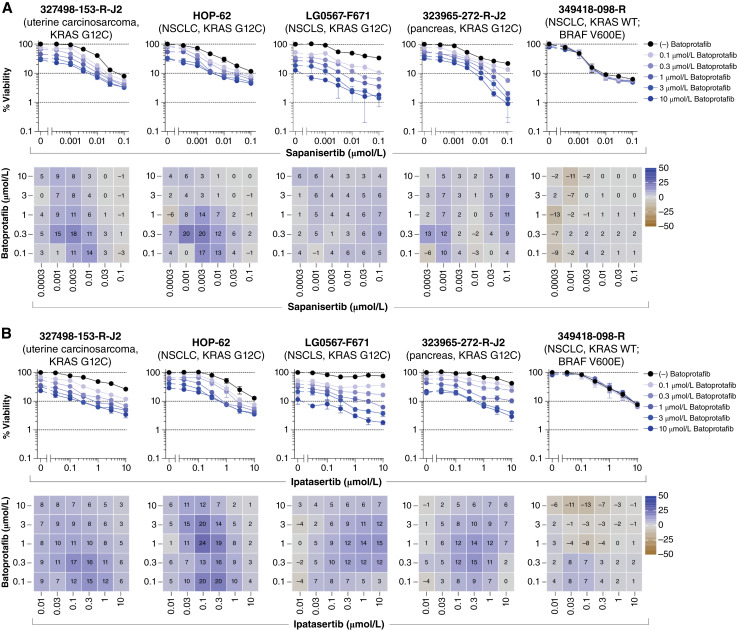
Dual pathway inhibition with batoprotafib in combination with sapanisertib or ipatasertib. Concentration–response graphs (top, mean ± SD, *n* = 4 technical replicates) from combinations of batoprotafib with (**A**) sapanisertib or (**B**) ipatasertib are shown, with corresponding Bliss independence scores from each combination’s concentration matrix (bottom, mean of *n* = 4 technical replicates) displayed numerically and as a heatmap (blue indicates synergy, gray indicates additivity, and brown indicates antagonism). The malignant cell line name, tumor type, and KRAS status are indicated above each set of graphs. WT, wild-type.

Targeting the PI3K/AKT/mTOR pathway with sapanisertib demonstrated both additive and synergistic activities in combination with batoprotafib in five models with the KRAS G12C variant (four of which are shown in Fig. 7A), whereas no combination activity was observed from the NSCLC model with wild-type KRAS and a BRAF V600E variant. Although most models without the KRAS G12C variant exhibited minimal responses to the combination of batoprotafib and sapanisertib, synergy was observed in the patient-derived colon model 186277-243-T-J2 with a KRAS G12D variant (Supplementary Fig. S11). Likewise, the combination of batoprotafib and ipatasertib yielded similar additive or synergistic activities to those seen with sapanisertib, demonstrating selective activity toward models with the KRAS G12C variant and the 186277-243-T-J2 model (Fig. 7B; Supplementary Fig. S12). Overall, the combinations of sapanisertib or ipatasertib with BI-3406 were less selective and effective compared with their combinations with batoprotafib (Supplementary Figs. S13 and S14).

**Figure 8.**
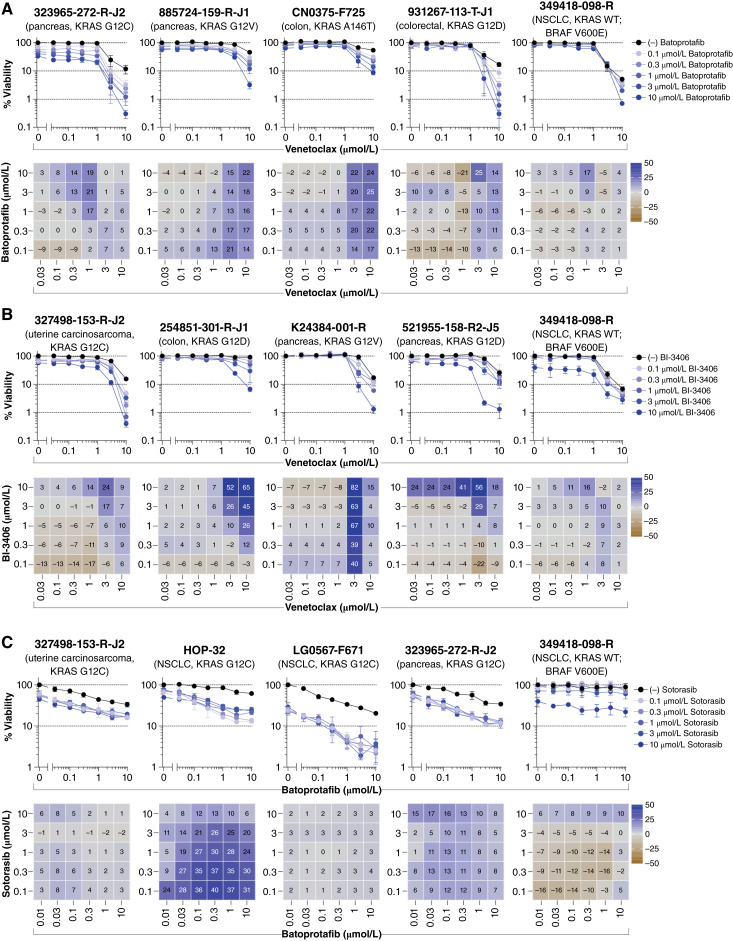
Combination of venetoclax with batoprotafib or BI-3406 and the combination of batoprotafib with sotorasib. Concentration–response graphs (top, mean ± SD, *n* = 4 technical replicates) from combinations of (**A**) batoprotafib or (**B**) BI-3406 with venetoclax, as well as the combination of (**C**) batoprotafib with sotorasib, are shown, with corresponding Bliss independence scores from each combination’s concentration matrix (bottom, mean of *n* = 4 technical replicates) displayed numerically and as a heatmap (blue indicates synergy, gray indicates additivity, and brown indicates antagonism). The malignant cell line name, tumor type, and KRAS status are indicated above each set of graphs. WT, wild-type.

Combinations of venetoclax with batoprotafib (Fig. 8A; Supplementary Fig. S15) or BI-3406 (Fig. 8B; Supplementary Fig. S16) also demonstrated notable activity. Greater-than-additive responses were primarily observed at higher concentrations of each agent. The combinations demonstrated additive and synergistic activity across the majority of KRAS variants but had limited impact on the 349418-098-R NSCLC model harboring wild-type KRAS and BRAF V600E. Lastly, concurrent inhibition of the KRAS pathway with the combination of sotorasib and batoprotafib resulted in both additive and synergistic activity in spheroids harboring the KRAS G12C variant (Fig. 8C). Most synergy was observed in the multicell-type tumor spheroids containing the HOP-62 NSCLC cell line, in which up to one log of cytotoxicity was achieved. Additive effects resulted in nearly 99% cytotoxicity (a two-log reduction) in the patient-derived NSCLC model LG0567-F671.

To investigate potential patterns of response among the 19 multicell-type tumor spheroids to the RAS pathway inhibitor combinations, a principal component analysis was performed. Data for the analysis incorporated both cell viability information and combination activity from the Bliss independence model. To summarize the cell viability of a tumor spheroid model throughout the entire concentration matrix of a drug combination, VUS values were calculated. The overall combination effects observed with a drug combination from a tumor spheroid model were described by the mean Bliss score that was calculated from the entire drug combination matrix. Although the analysis revealed four distinct clusters of tumor spheroid models based on their *in vitro* responses (Supplementary Fig. S17), no obvious relationships were identified between KRAS status and sensitivity to the various combinations.

## Discussion

*KRAS* is widely recognized as one of the most frequently altered oncogenes in cancer. A recent analysis of The Cancer Genome Atlas database showed that *KRAS* variants are present in nearly 12% of all cancers, with PDAC showing the highest prevalence at more than 80%, followed by colorectal cancer at nearly 40%, and approximately 20% in patients with NSCLC (50). The landmark FDA approval of sotorasib for the treatment of NSCLC bearing the KRAS G12C variant proved that exquisite targeting of malignant cells can have a therapeutic benefit (27). Approximately 1 year later, the KRAS G12C inhibitor adagrasib received accelerated approval from the FDA for the same indication (51). A range of additional KRAS G12C inhibitors are currently under development and include olomorasib (LY3537982), divarasib (GDC-6036), garsorasib (D-1553), HBI-2438, opnurasib (JDQ443), glecirasib (JAB-21822), HS-10370, IBI351 (GFH925), BI 1823911, JNJ-74699157, ARS-853, ARS-1620, ASP2453, and ERAS-3490 (6). Inhibitors targeting other KRAS variants, including G12D, G12S, and G12R, are also under development (52–54), with several G12D-selective inhibitors now progressing through clinical trials (55). Beyond directly targeting specific KRAS variants, the development of pan-KRAS(ON) inhibitors that target the full spectrum of KRAS variants shows promise for effectively treating a range of cancers (56–58). It has recently been noted that sotorasib is an effective inhibitor not only of KRAS G12C but also of HRAS G12C and NRAS G12C, expanding the range and number of tumors in which sotorasib might be therapeutically important (59). Although sotorasib treatment resulted in significant responses in the targeted patient population, durable complete responses were not achieved. Improved treatment for patients with KRAS G12C bearing NSCLC will likely come from combination therapeutic regimens, including immunotherapy, radiotherapy, and chemotherapy.

There have also been efforts to suppress RAS pathway activity by targeting signaling upstream of RAS. Two such efforts include targeting SHP2 and SOS1, both of which disrupt the RAS nucleotide exchange process (60). Clinical trials of SHP2 and SOS1 inhibitors as monotherapy have demonstrated limited efficacy, so drug combinations are under clinical evaluation (61–63). SHP2 has been shown to regulate immunologic checkpoints and inhibit antitumor immune responses (64, 65). Consequently, several clinical trials are evaluating the combination of SHP2 inhibitors with immunotherapy (clinicaltrials.gov; NCT05375084, NCT05505877, NCT04000529).

In this study, the allosteric SHP2 inhibitor batoprotafib, the SOS1 inhibitor BI-3406, and the KRAS G12C allele–specific inhibitor sotorasib were examined in combination with a range of targeted small-molecule agents (Supplementary Table S1). Potential approaches explored for combination chemotherapy regimens included targeting RTKs (erdafitinib, nintedanib, cabozantinib), mitogen-activated protein kinases (trametinib, temuterkib), PI3K/AKT/mTOR signaling pathway intermediates (ipatasertib, sapanisertib), or complementary targets such as BCL-2 (venetoclax) or PARP (olaparib, talazoparib). Single agents and combinations were evaluated in multicell-type tumor spheroid models grown from 19 malignant cell lines derived from cancers with a range of oncogenic KRAS variants (Supplementary Table S2).

As a single agent, sotorasib showed the most activity in two patient-derived lung adenocarcinoma models with KRAS G12C, 941728-121-R-J1 and LG0567-F671 (Fig. 1; Supplementary Figs. S1A and S2A). The 941728-121-R-J1 cell line has a *KRAS* copy number of 4.6, loss of heterozygosity, and a variant allele frequency of 1.0, whereas the LG0567-F671 cell line has a *KRAS* copy number of 2.0, no loss of heterozygosity, and a variant allele frequency of 0.42 (Supplementary Fig. S2A; Supplementary Table S2). Therefore, the differential responses of the models might be attributed in part to genetic differences.

As single agents, sotorasib and batoprotafib demonstrated selectivity toward tumor spheroids harboring KRAS G12C, whereas BI-3406 elicited responses in a broader range of models (Fig. 1A and B). However, previous studies have shown that cell lines expressing KRAS G12C or G12A demonstrated selective sensitivity to the allosteric SHP2 inhibitor RMC-4550 (66). The activity of SHP2 inhibitors correlates with KRAS GTPase activity (67, 68). This can partly be attributed to the differential impact of KRAS variants on GTPase activity or GAP-mediated hydrolysis, wherein G12C compared with other variants, demonstrated an intrinsic GTP hydrolysis rate nearly equivalent to that of wild-type KRAS (69). Conversely, SOS1 inhibitors disrupt the protein–protein interaction between SOS1 and KRAS by binding to the SOS1 catalytic site, preventing nucleotide exchange and KRAS GTP loading. This mechanism suggests their potential efficacy against a broad range of KRAS variants (34, 70).

Efforts to target downstream effectors of RAS have involved inhibitors of BRAF, MEK, and ERK (71–73). However, these strategies have faced challenges from compensatory feedback mechanisms, which can lead to the reactivation of multiple RTKs and the emergence of therapeutic resistance (74). Both SOS1 and SHP2 function as proximal signaling intermediates for RTKs upstream of RAS and are essential for RTK-dependent RAS activation and subsequent pathway feedback mechanisms. Among the drug combinations evaluated in this study, combination effects were frequently observed from concurrently blocking a downstream effector and an upstream activator of KRAS, irrespective of KRAS status. For instance, vertical pathway inhibition with the SHP2 inhibitor batoprotafib in combination with either the MEK inhibitor trametinib or the ERK inhibitor temuterkib led to additive and/or synergistic responses (Fig. 4; Supplementary Figs. S4 and S5). Similar results were observed with the SOS1 inhibitor BI-3406 (Fig. 5; Supplementary Figs. S7 and S8). The consistency of these effects across the tumor spheroid models is indicated by correlations in mean Bliss scores (Supplementary Fig. S6) and reflects the similar point at which SHP2 and SOS1 inhibitors disrupt the RAS signaling pathway. In preclinical studies, the combination of BI-3406 and the MEK inhibitor selumetinib reduced neurofibroma cell proliferation and tumor volumes in a mouse model of plexiform neurofibroma (75). Additionally, a combination of the SOS1 inhibitor SGR-4174 and trametinib demonstrated activity *in vitro* and *in vivo* (76). Recently SHP2 inhibition was identified as a vulnerability in NSCLC harboring a KRAS variant and synergy between SHP2 and MEK inhibitors was observed in multiple NSCLC cell lines with KRAS variants (77). Similarly, combination activity was observed from batoprotafib and trametinib in NSCLC multicell-type tumor spheroid models harboring KRAS variants in the current study (Fig. 4A; Supplementary Fig. S4). Several clinical trials are underway to evaluate combinations of a MEK inhibitor with the SOS1 inhibitor BI 1701963 (closely related to BI-3406) or a SHP2 inhibitor [clinicaltrials.gov; NCT03989115, NCT04292119, NCT04800822 (78), and NCT04111458 (79)]. Combinations of a SHP2 inhibitor with an ERK inhibitor are also undergoing evaluation in the clinic [clinicaltrials.gov; NCT04866134 (80) and NCT04916236 (81)]. Although the combination activity of sotorasib with either trametinib or temuterkib (Fig. 2A and B) was modest compared with the more pronounced activity observed with batoprotafib (Fig. 4; Supplementary Figs. S4 and S5) or BI-3406 (Fig. 5; Supplementary Figs. S7 and S8), antitumor activity was observed from the combination of sotorasib and trametinib, including responses in patients previously treated with KRAS G12C inhibitors (82). Ongoing clinical trials continue to explore downstream targets of RAS in combination with KRAS G12C inhibitors.

Both SHP2 and SOS1 inhibitors exhibited cytotoxicity in RTK-driven human cancer cells, suggesting that inhibitors targeting oncogenic RTKs upstream of RAS might sensitize cells to SHP2 or SOS1 inhibition (38, 83). The combined pharmacologic inhibition of SHP2 or SOS1 with EGFR variant–specific inhibitors, such as nazartinib and osimertinib, produced synergistic effects in both NSCLC cell lines and patient-derived xenografts with an EGFR variant (83–85). In addition, blocking SHP2 in conjunction with an FGFR-targeted kinase inhibitor synergistically inhibited the growth of metastatic breast cancer cells and suppressed metastatic progression *in vivo* (86). In the multicell-type tumor spheroids, combinations of batoprotafib or BI-3406 with nintedanib, a multitargeted RTK inhibitor, demonstrated additive and synergistic responses at the higher concentrations of each agent and across KRAS variants (Fig. 6). Several clinical trials are currently assessing the combination of SHP2 inhibitors with RTK inhibitors, including anlotinib, a multitargeted RTK inhibitor (clinicaltrials.gov; NCT05715398), as well as two EGFR inhibitors, nazertinib (clinicaltrials.gov; NCT03114319) and osimertinib (clinicaltrials.gov; NCT03989115). The most substantial combination effects for sotorasib with nintedanib were observed in the KRAS G12C–containing pancreatic 323965-272-R-J2 and uterine carcinosarcoma 327498-153-R-J2 tumor spheroid models (Fig. 2C). Clinical results from combining sotorasib with the EGFR inhibitor afatinib in patients with NSCLC demonstrated overall response rates of 20% and 34.8%, with disease control rates of 70% and 73.9% across two dose cohorts (87). Additionally, combining KRAS G12C inhibitors with anti-EGFR monoclonal antibodies has shown encouraging antitumor activity compared with the standard of care in colorectal cancer (88–91).

The RAS/MEK/ERK and PI3K/AKT/mTOR pathways continue to be pivotal targets for anticancer therapies (6, 92). In preclinical studies, simultaneous inhibition of both pathways demonstrated greater efficacy than targeting a single pathway. However, translating this approach to human patients has been challenging due to dose-limiting toxicities and the difficulty in establishing a therapeutic window (93–95). Combination effects were apparent in the multicell-type tumor spheroids when pairing batoprotafib with the PI3K/AKT/mTOR pathway inhibitors sapanisertib (Fig. 7A; Supplementary Fig. S11) or ipatasertib (Fig. 7B; Supplementary Fig. S12). Notably, these combinations displayed relatively selective activity toward tumor spheroids harboring KRAS G12C. A recent study reported synergistic effects from the combined pharmacologic inhibition of SHP2 and AKT on the growth of five colorectal carcinoma cell lines, as well as substantial reductions in xenograft tumor volumes compared with the single agents (96). In the current study, synergy was observed between batoprotafib and ipatasertib in the 186277-243-T-J2 colon adenocarcinoma tumor spheroids (KRAS G12D), which achieved two logs of cytotoxicity at the highest concentrations of both agents. However, an IC_90_ was not reached in the other six colorectal spheroid models (Supplementary Fig. S12). In comparison with batoprotafib, the combination of BI-3406 with sapanisertib (Supplementary Fig. S13) or ipatasertib (Supplementary Fig. S14) showed similar activity and selectivity. Similar outcomes were observed with the concurrent inhibition of SHP2 and the PI3K/AKT/mTOR pathway from *in vitro* and *in vivo* models of liver, ovarian, and metastatic breast cancer (97–99). Dual pathway inhibition by sotorasib combined with either sapanisertib (Fig. 3A) or ipatasertib (Fig. 3B) also exhibited combination activity. Targeting the PI3K/AKT/mTOR pathway has been shown to be effective when combined with KRAS G12C inhibitors (22, 41, 100). For example, sotorasib in combination with an mTORC1/2 inhibitor led to sustained inhibition of tumor growth and metastasis in a mouse model of PDAC (101). There are two clinical trials investigating the combination of KRAS G12C inhibitors with mTOR inhibitors in solid tumors harboring KRAS G12C (clinicaltrials.gov; NCT05840510 and NCT04185883).

Combination effects were also observed in various spheroid models when the BCL-2 inhibitor venetoclax was combined with batoprotafib (Fig. 8A; Supplementary Fig. S15), BI-3406 (Fig. 8B; Supplementary Fig. S16), or sotorasib (Fig. 3C). Although responses from the combination of sotorasib with venetoclax were primarily additive in models harboring KRAS G12C, greater-than-additive responses were observed from combinations of venetoclax with batoprotafib and BI-3406. Recently, synergy between the allosteric SHP2 inhibitor RMC-4550 and venetoclax was observed in acute myeloid leukemia models with alterations in *FLT3* and *KIT*, indicating that SHP2 inhibition enhances cellular dependence on BCL-2 (102). The combination of a SOS1 inhibitor with venetoclax improved efficacy in an acute myeloid leukemia cell line and patient-derived xenograft models compared with either agent alone (103). In clinical settings, BCL-2 inhibitors seem to have less efficacy against solid tumors compared with hematologic cancers when used as single-agent therapy (104). Results from the multicell-type tumor spheroid models suggest that KRAS, SHP2, or SOS1 inhibitors could enhance the effectiveness of BCL-2 inhibitors against solid tumors; however, hematologic toxicity might be a limitation.

The combination of sotorasib with batoprotafib exhibited both additive and synergistic responses in KRAS G12C tumor spheroids (Fig. 8C). Mechanistically, inhibiting SHP2 or SOS1 reduces the GDP–GTP exchange rate and increases the occupancy of the KRAS G12C-GDP state, which is targeted by some KRAS G12C inhibitors (12). Multiple clinical trials are exploring combinations of KRAS G12C inhibitors with SHP2 or SOS1 inhibitors (105–108). Recent clinical results suggest promising outcomes for this combination strategy. For instance, the combination of the KRAS G12C inhibitor glecirasib with the SHP2 inhibitor JAB-3312 demonstrated an overall response rate of 50% (14/28) and a disease control rate of 100% in patients with KRAS G12C inhibitor-naive NSCLC (109).

Altogether, the findings in these patient-derived multicell-type tumor spheroid models confirm and broaden the understanding of drug combinations with inhibitors of KRAS G12C, SHP2, and SOS1. Many of the combination strategies evaluated in this study are undergoing investigation in clinical trials. Continued research will be essential to validate the efficacy of these combination strategies and their potential for cancer therapy.

## Supplementary Material

Figure S1 Figure S1. Response to sotorasib by multicell-type tumor spheroid models.

Figure S2 Figure S2. Response to sotorasib by tumor models harboring KRAS G12C.

Figure S3 Figure S3. Combination activity of selected targeted agents with sotorasib in spheroids grown from HUVEC and hMSC.

Figure S4 Figure S4. Combination activity of batoprotafib with trametinib in multicell-type tumor spheroids.

Figure S5 Figure S5. Combination activity of batoprotafib with temuterkib in multicell-type tumor spheroids.

Figure S6 Figure S6. Mean Bliss score correlations for vertical inhibition of the KRAS pathway by batoprotafib or BI-3406 in combination with trametinib or temuterkib.

Figure S7 Figure S7. Combination activity of BI-3406 with trametinib in multicell-type tumor spheroids.

Figure S8 Figure S8. Combination activity of BI-3406 with temuterkib in multicell-type tumor spheroids.

Figure S9 Figure S9. Combination activity of batoprotafib with nintedanib in multicell-type tumor spheroids.

Figure S10 Figure S10. Combination activity of BI-3406 with nintedanib in multicell-type tumor spheroids.

Figure S11 Figure S11. Combination activity of batoprotafib with sapanisertib in multicell-type tumor spheroids.

Figure S12 Figure S12. Combination activity of batoprotafib with ipatasertib in multicell-type tumor spheroids.

Figure S13 Figure S13. Combination activity of BI-3406 with sapanisertib in multicell-type tumor spheroids.

Figure S14 Figure S14. Combination activity of BI-3406 with ipatasertib in multicell-type tumor spheroids.

Figure S15 Figure S15. Combination activity of batoprotafib with venetoclax in multicell-type tumor spheroids.

Figure S16 Figure S16. Combination activity of BI-3406 with venetoclax in multicell-type tumor spheroids.

Figure S17 Figure S17. Sensitivity profiles of the 19 multicell-type tumor spheroids from all RAS pathway inhibitor combinations.

Table S1 Table S1. Drugs and investigational agents used in this study. The agents included RAS pathway inhibitors (top) and molecular targeted combination drugs (bottom). If available at the time of this study, the clinical Cmax is listed.

Table S2 Table S2. The malignant cell lines grown as multicell-type tumor spheroids for this study. The names of both patient-derived and established cell lines are listed along with the tumor type they were derived from and the KRAS status.

Table S3 Table S3. Cell inoculation densities for multicell-type tumor spheroid models per well in 384-well microplates.

Table S4 Table S4. The variant status of key signaling proteins from the NCI PDMR patient-derived cancer cell lines.

Table S5 Table S5. Gene copy number for key signaling proteins from the NCI PDMR patient-derived cancer cell lines.

Table S6 Table S6. Oncoprint

Supplementary Materials & Methods Supplementary Materials & Methods

## Data Availability

All data are accessible via the PubChem BioAssay public database (AID 1963875; AID 1963874; AID 1963868; AID 1963873; AID 1963870; AID 1963869; AID 1963867; AID 1963866; AID 1963872; AID 1963871; AID 1963864; AID 1963865; AID 1963861; AID 1963863; AID 1963862; AID 1963860; AID 1963858; AID 1963859; AID 1963857; AID 1963856). The PDC cell line data used in this study can be downloaded from the NCI PDMR.
