# Supplementary material for: RAS Pathway Inhibitors Combined with Targeted Agents Are Active in Patient-Derived Spheroids with Oncogenic KRAS Variants from Multiple Cancer Types
Source: Cancer Res Commun. 2025 Oct 8;5(10):1779–95. doi: 10.1158/2767-9764.CRC-24-0582 (PMC12505081; doi:10.1158/2767-9764.CRC-24-0582)
Supplement: Figure S1 — Response to sotorasib by multicell-type tumor spheroid models. [file crc-24-0582_figure_s1_suppsf1.pdf]

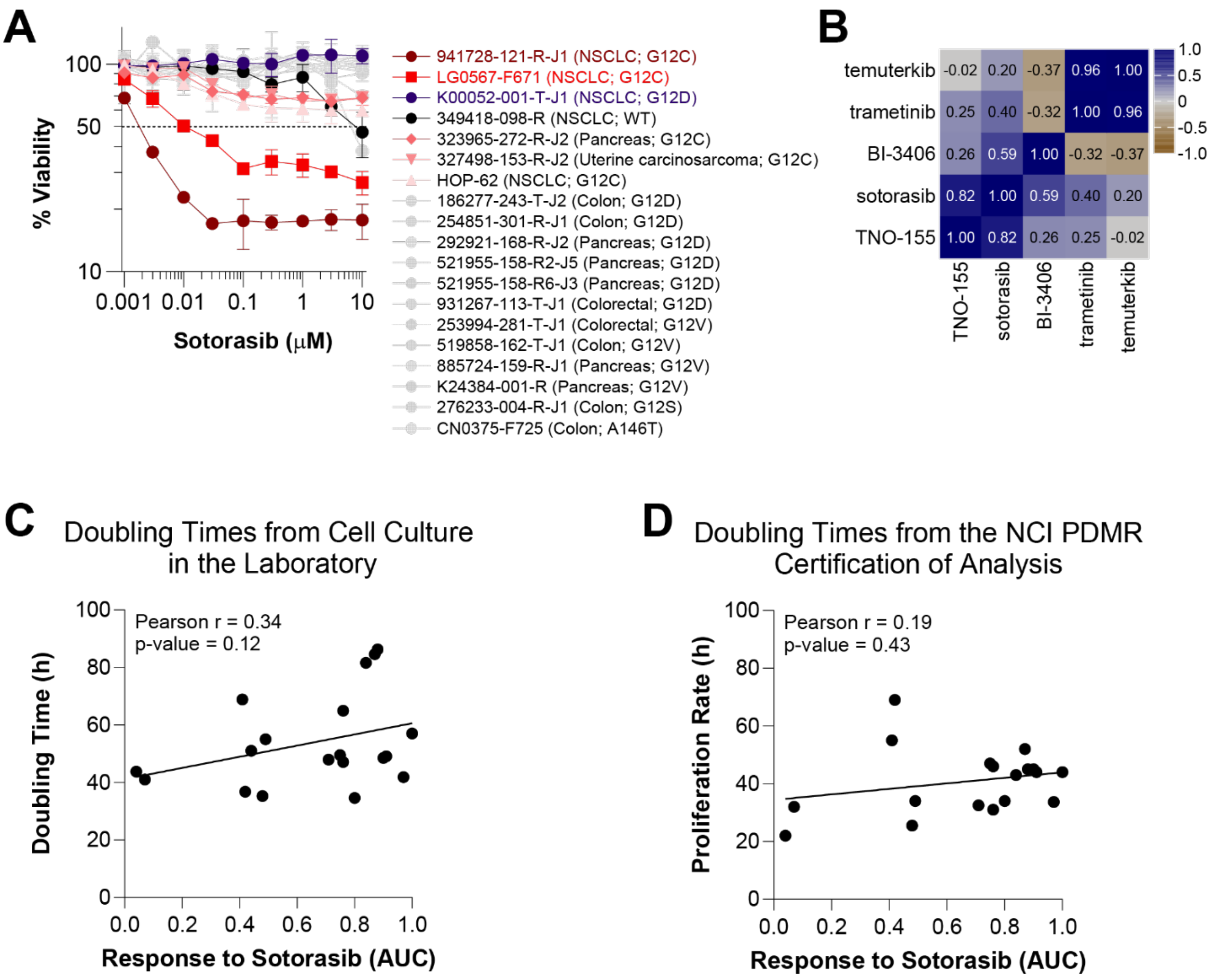

Figure S1. **Response to sotorasib by multicell-type tumor spheroid models**. (A) Concentration-response graphs (mean ± SD, n = 6 technical replicates) of nineteen multicell-type tumor spheroid models treated with nine concentrations of sotorasib. (B) Pearson's correlation matrix of the Area Under the Curve responses by spheroid models harboring KRAS G12C (n = 5) to the five RAS pathway inhibitors. Only the correlation between trametinib and temuterkib achieved statistical significance (Pearson r = 0.96, p = 0.009). (C) No correlation was observed between the response to sotorasib by area under the curve (AUC) and the doubling time of cell lines, either obtained from laboratory culturing (Pearson r = 0.34, p = 0.12) or (D) from the NCI PDMR Certification of Analysis (Pearson r = 0.19, p = 0.43).
