# Supplementary material for: RAS Pathway Inhibitors Combined with Targeted Agents Are Active in Patient-Derived Spheroids with Oncogenic KRAS Variants from Multiple Cancer Types
Source: Cancer Res Commun. 2025 Oct 8;5(10):1779–95. doi: 10.1158/2767-9764.CRC-24-0582 (PMC12505081; doi:10.1158/2767-9764.CRC-24-0582)
Supplement: Figure S2 — Response to sotorasib by tumor models harboring KRAS G12C. [file crc-24-0582_figure_s2_suppsf2.pdf]

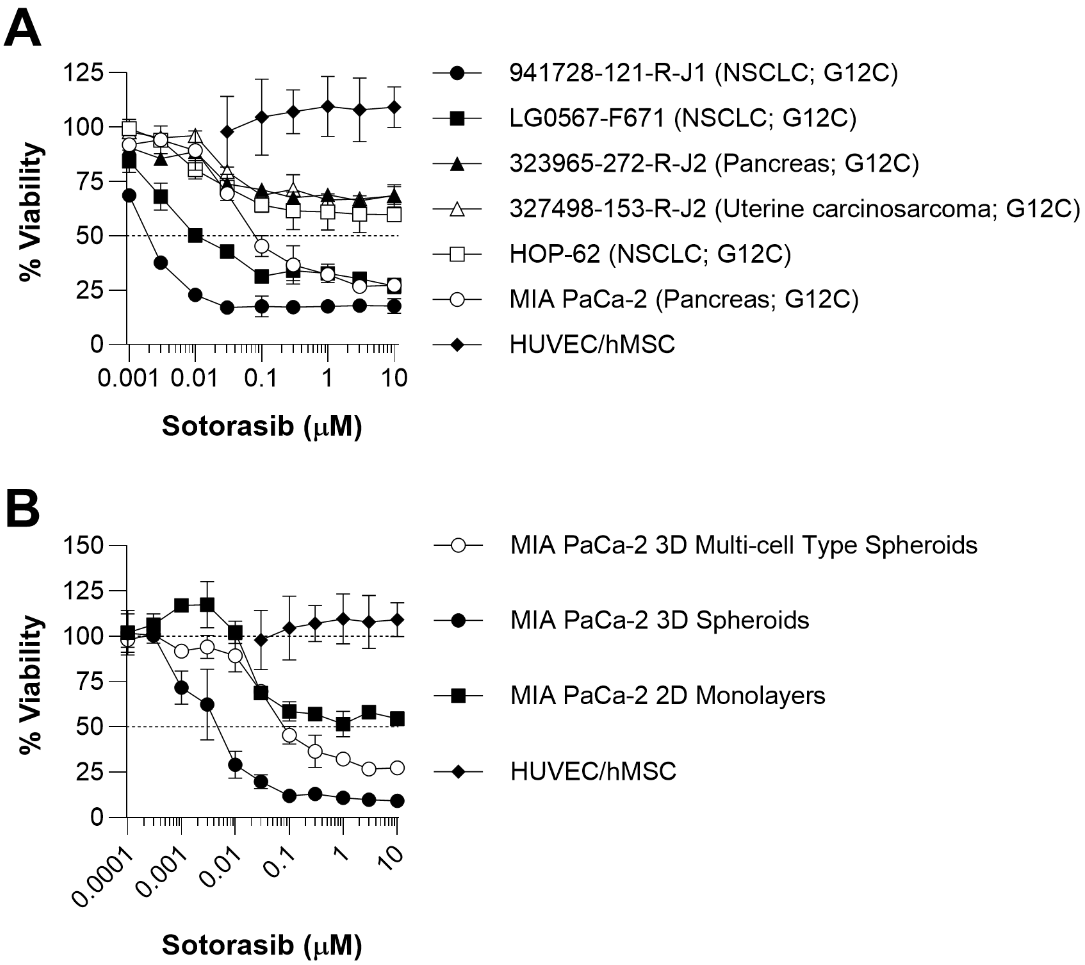

Figure S2. **Response to sotorasib by tumor models harboring KRAS G12C**. (A) Concentration-response graphs (mean ± SD, *n* = 6 technical replicates except for MIA PaCa-2 with *n* = 3) of multicell-type tumor spheroid models treated with nine concentrations of sotorasib. In addition to the five models featured in this study that harbor KRAS G12C, data are shown from MIA PaCa-2 cells (pancreas; G12C) grown as multicell-type tumor spheroids. Data are also shown from HUVEC and hMSC grown as spheroids and treated with six concentrations of sotorasib (*n* = 3 technical replicates). The 941728-121-R-J1 cell line has a KRAS copy number = 4.6, loss of heterozygosity (LOH) and variant allele frequency (VAF) = 1.0 (446 variant reads out of 446 total). The LG0567-F671 cell line has a KRAS copy number = 2.0, no LOH, and VAF = 0.42 (60 variant reads out of 144 total). The 323965-272-R-J2 cell line has a KRAS copy number = 2.1, no LOH, and VAF = 0.62 (164 variant reads out of 265 total). The 327498-153-R-J2 cell line has a KRAS copy number = 2.9, no LOH, and VAF = 0.30 (89 variant reads out of 293 total). (B) MIA PaCa-2 cells grown either as single-cell type monolayers or spheroids as well as multicell-type tumor spheroids with HUVEC and hMSC were treated with nine concentrations of sotorasib (mean ± SD, *n* = 3 technical replicates). Prior to the viability endpoint, all models were exposed to sotorasib or vehicle for 7 days except the monolayer, which had a 3-day exposure period (see the Supplementary Materials and Methods). Data are also shown from HUVEC and hMSC grown as spheroids and treated with six concentrations of sotorasib (*n* = 3 technical replicates).
