# Supplementary material for: RAS Pathway Inhibitors Combined with Targeted Agents Are Active in Patient-Derived Spheroids with Oncogenic KRAS Variants from Multiple Cancer Types
Source: Cancer Res Commun. 2025 Oct 8;5(10):1779–95. doi: 10.1158/2767-9764.CRC-24-0582 (PMC12505081; doi:10.1158/2767-9764.CRC-24-0582)
Supplement: Figure S3 — Combination activity of selected targeted agents with sotorasib in spheroids grown from HUVEC and hMSC. [file crc-24-0582_figure_s3_suppsf3.pdf]

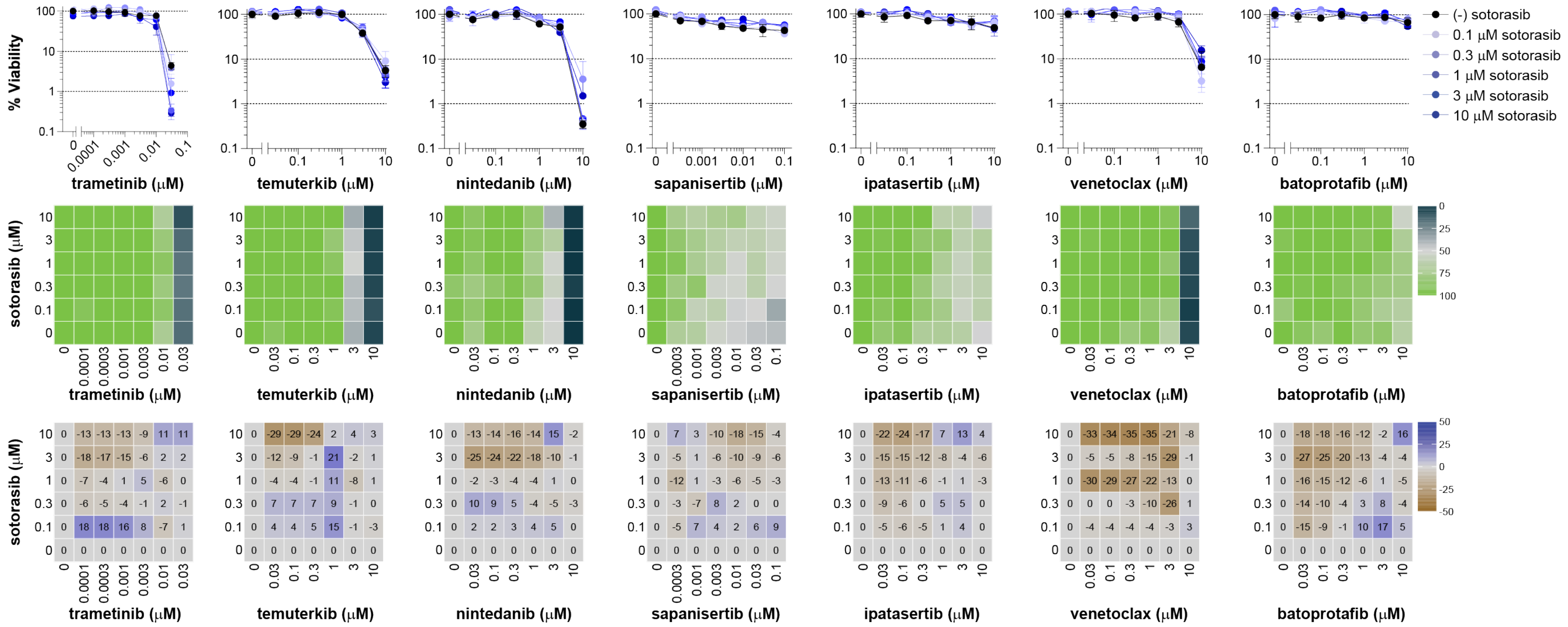


**Figure S3**. **Combination activity of selected targeted agents with sotorasib in spheroids grown from HUVEC and hMSC**. Concentration-response graphs (*top*, mean ± SD, *n* = 3 technical replicates), % viability across the combination's concentration matrix (*middle*, mean of *n* = 3 technical replicates) displayed as a heatmap (green indicates high cell viability and black indicates low cell viability), and Bliss independence scores across the combination's concentration matrix (*bottom*, mean of *n* = 3 technical replicates) displayed numerically and as a heatmap (blue indicates synergy, gray indicates additivity, and brown indicates antagonism). Data are shown from multicell-type spheroids grown from HUVEC and hMSC following exposure to sototorasib as a single agent and in combination with trametinib, temuterkib, nintedanib, sapanisertib, ipatasertib, venetoclax, and batoprotafib.
