# Supplementary figures and images for: RAS Pathway Inhibitors Combined with Targeted Agents Are Active in Patient-Derived Spheroids with Oncogenic KRAS Variants from Multiple Cancer Types
Source: Cancer Res Commun. 2025 Oct 8;5(10):1779–95. doi: 10.1158/2767-9764.CRC-24-0582 (PMC12505081; doi:10.1158/2767-9764.CRC-24-0582)

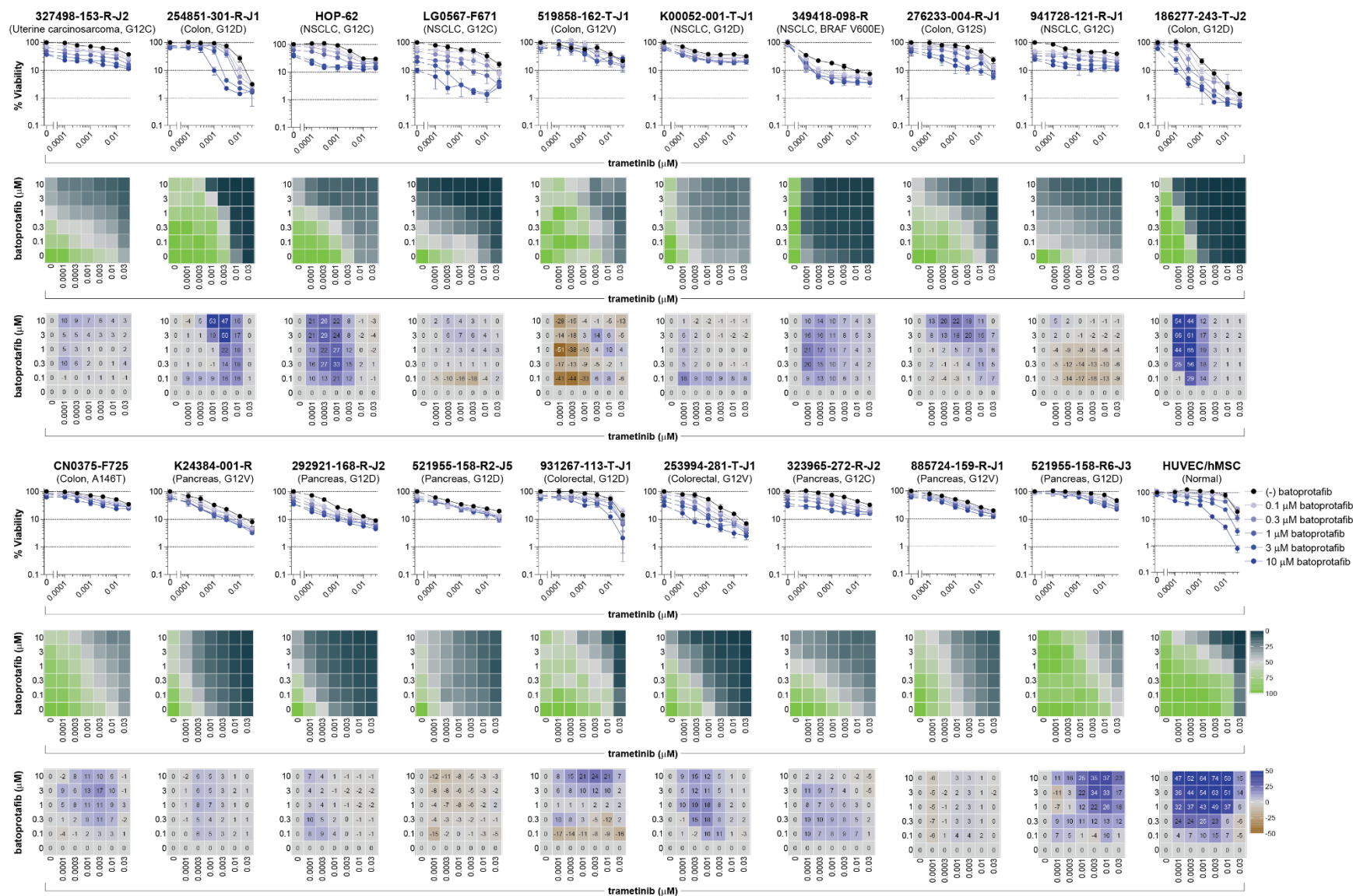

Supplement: Figure S4 — Combination activity of batoprotafib with trametinib in multicell-type tumor spheroids. [file crc-24-0582_figure_s4_suppsf4.pdf]

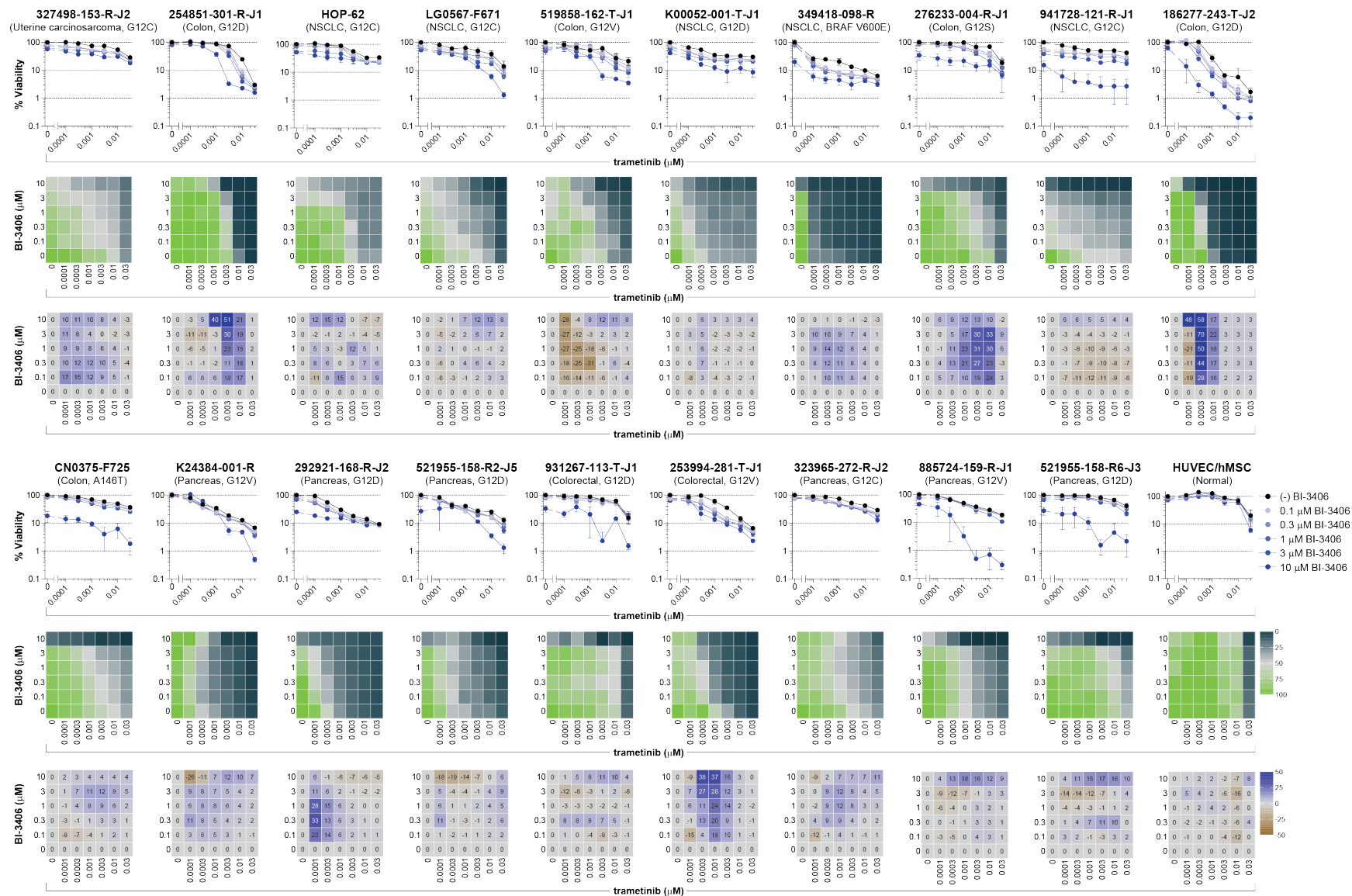

Supplement: Figure S7 — Combination activity of BI-3406 with trametinib in multicell-type tumor spheroids. [file crc-24-0582_figure_s7_suppsf7.pdf]

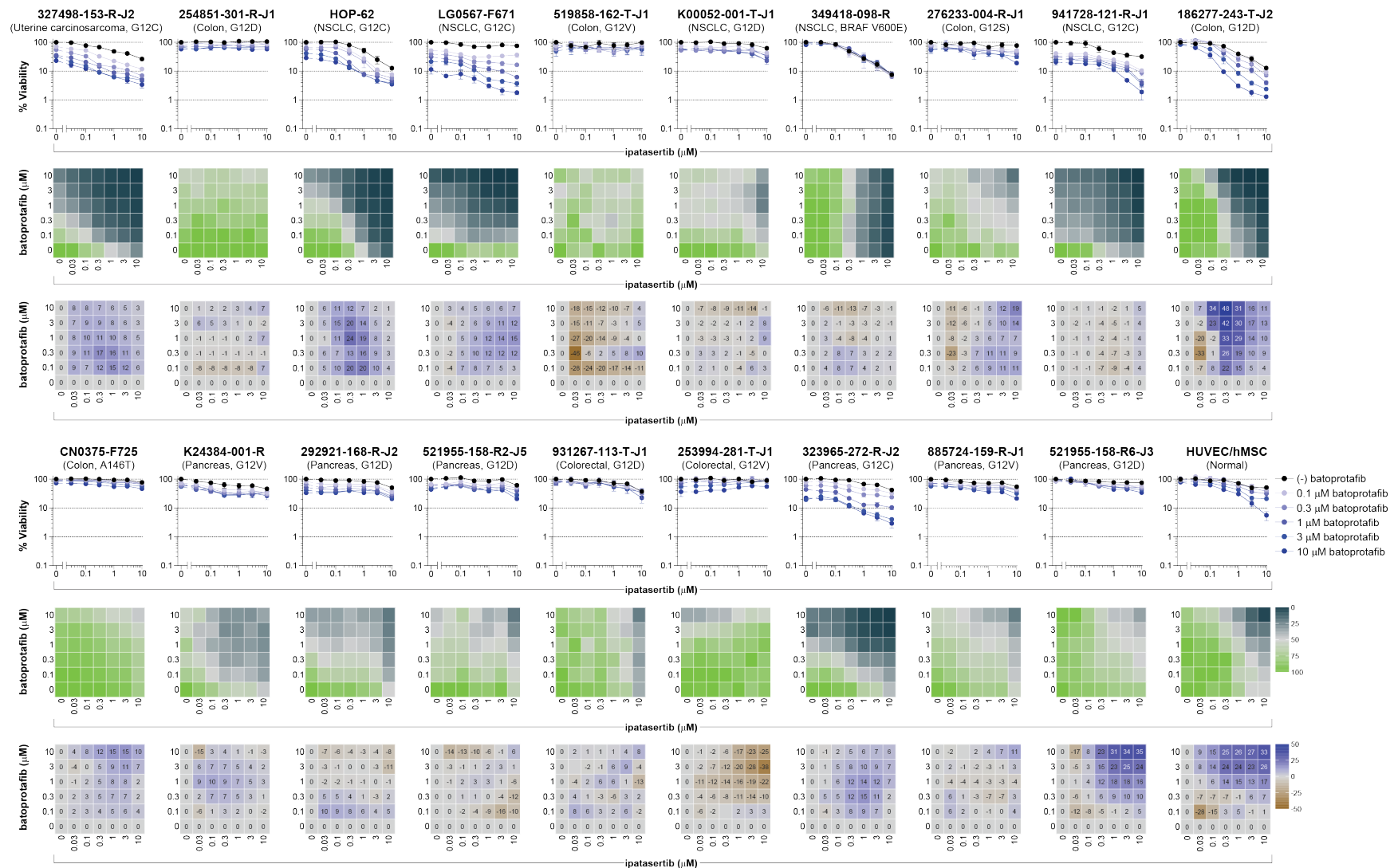

Supplement: Figure S12 — Combination activity of batoprotafib with ipatasertib in multicell-type tumor spheroids. [file crc-24-0582_figure_s12_suppsf12.pdf]

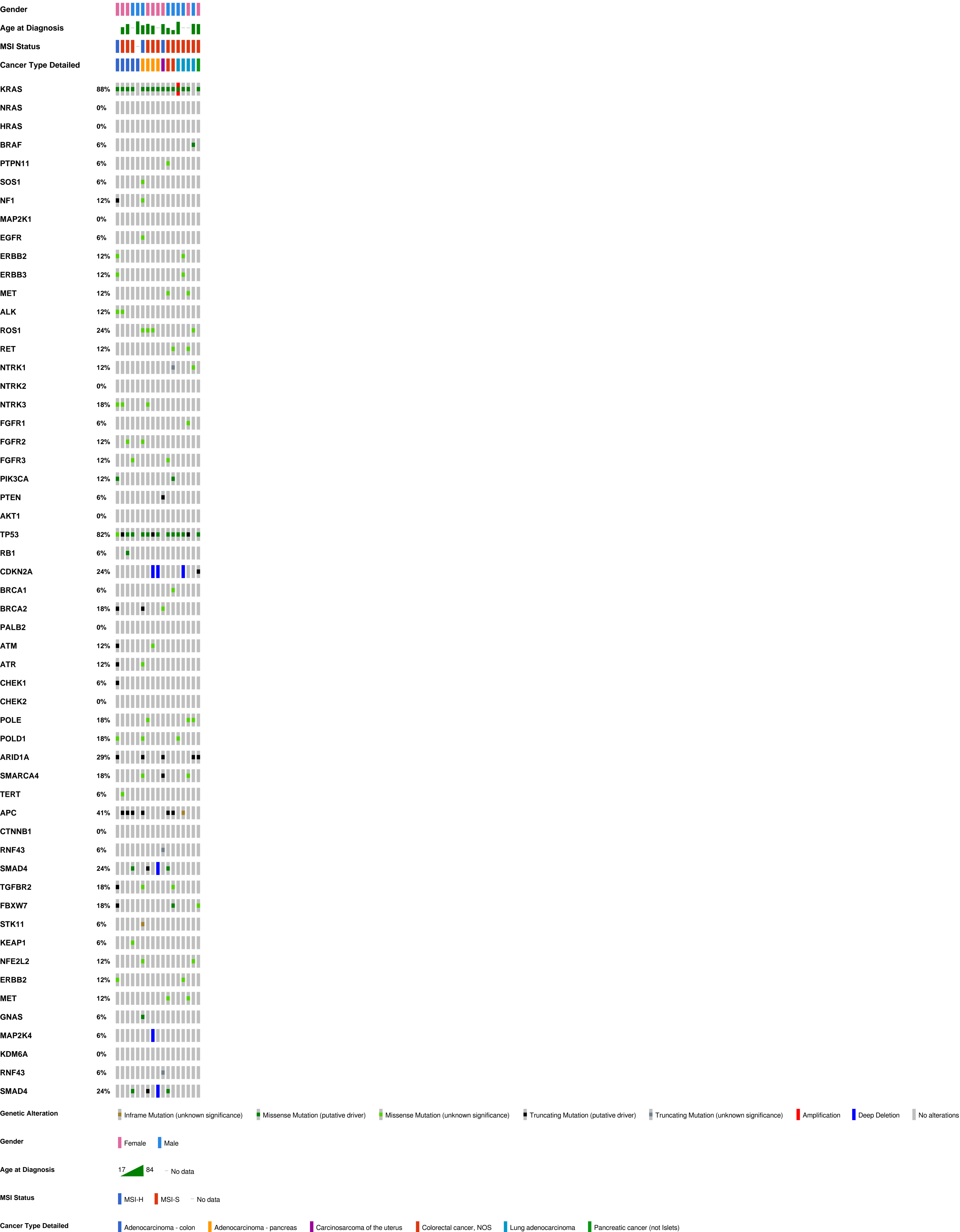

Supplement: Table S6 — Oncoprint [file crc-24-0582_table_s6_suppst6.pdf]
