# Supplementary material for: RAS Pathway Inhibitors Combined with Targeted Agents Are Active in Patient-Derived Spheroids with Oncogenic KRAS Variants from Multiple Cancer Types
Source: Cancer Res Commun. 2025 Oct 8;5(10):1779–95. doi: 10.1158/2767-9764.CRC-24-0582 (PMC12505081; doi:10.1158/2767-9764.CRC-24-0582)
Supplement: Figure S5 — Combination activity of batoprotafib with temuterkib in multicell-type tumor spheroids. [file crc-24-0582_figure_s5_suppsf5.pdf]

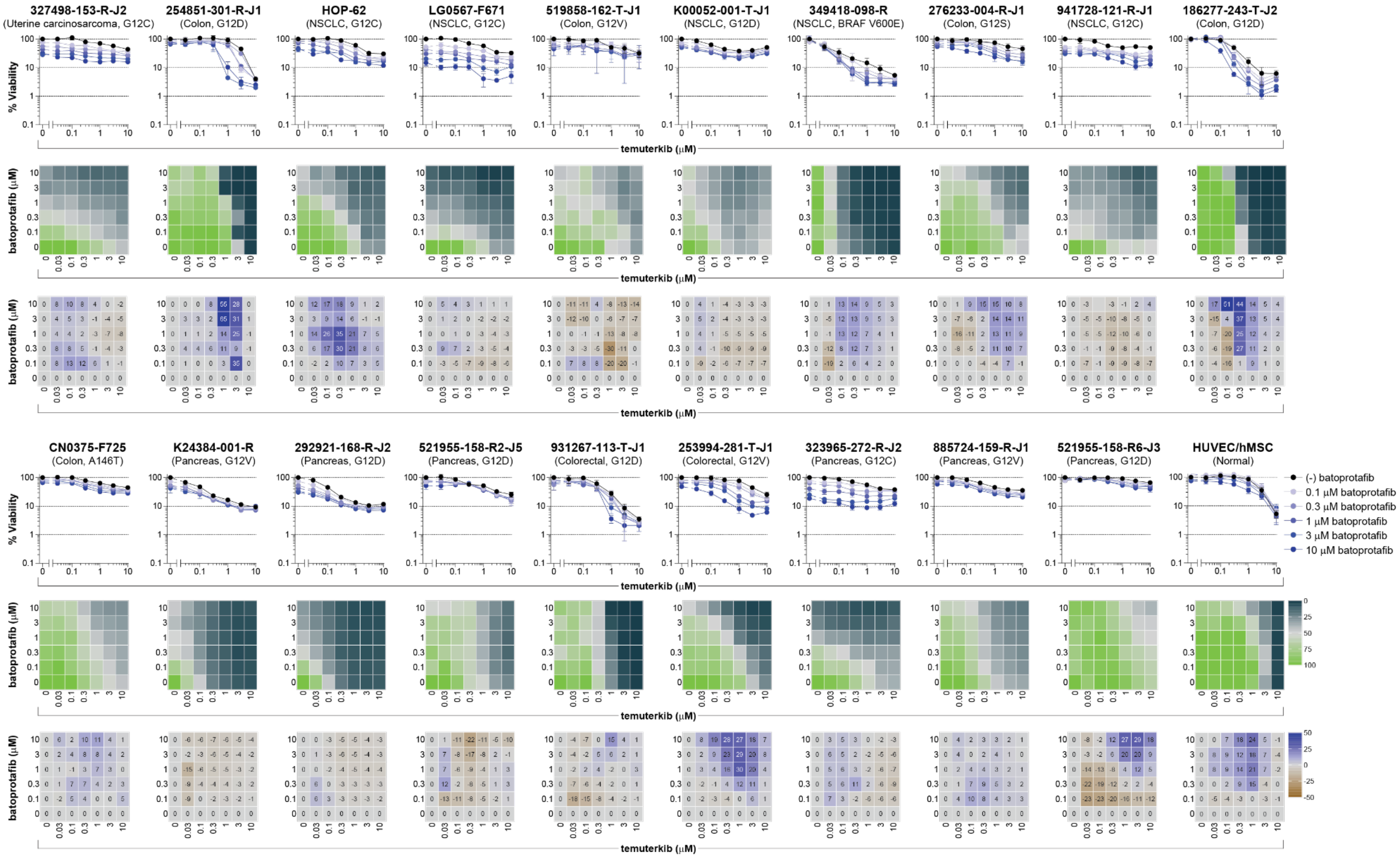

Figure S5. **Combination activity of batoprotafib with temuterkib in multicell-type tumor spheroids**. Concentration-response graphs (*top*, mean ± SD, n = 4 technical replicates), % viability across the combination's concentration matrix (*middle*, mean of n = 4 technical replicates) displayed as a heatmap (green indicates high cell viability and black indicates low cell viability), and Bliss independence scores across the combination's concentration matrix (*bottom*, mean of n = 4 technical replicates) displayed numerically and as a heatmap (blue indicates synergy, gray indicates additivity, and brown indicates antagonism). Data are shown from nineteen malignant cell lines grown as multicell-type tumor spheroids with HUVEC and hMSC following exposure to batoprotafib and temuterkib as single agents and in combinations. Data are also shown from HUVEC and hMSC grown as spheroids (mean of n = 3 technical replicates). The malignant cell line name, tumor type, and KRAS status are indicated above each set of graphs.
