# Supplementary material for: RAS Pathway Inhibitors Combined with Targeted Agents Are Active in Patient-Derived Spheroids with Oncogenic KRAS Variants from Multiple Cancer Types
Source: Cancer Res Commun. 2025 Oct 8;5(10):1779–95. doi: 10.1158/2767-9764.CRC-24-0582 (PMC12505081; doi:10.1158/2767-9764.CRC-24-0582)
Supplement: Figure S6 — Mean Bliss score correlations for vertical inhibition of the KRAS pathway by batoprotafib or BI-3406 in combination with trametinib or temuterkib. [file crc-24-0582_figure_s6_suppsf6.pdf]

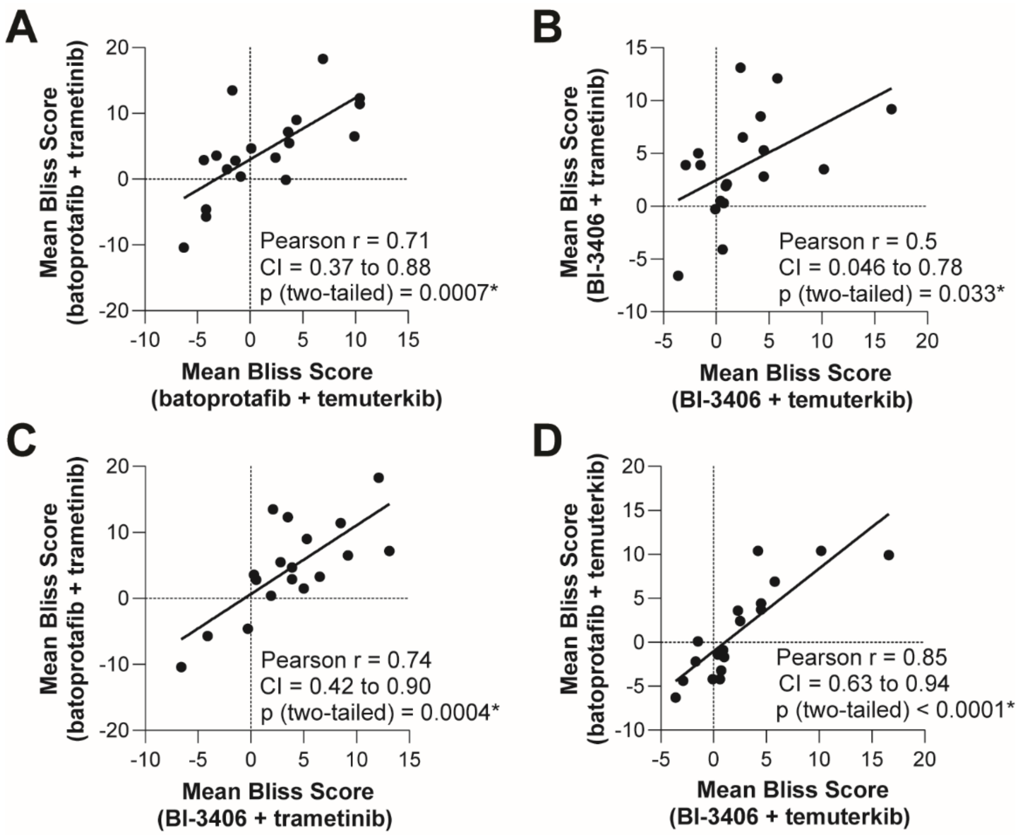

Figure S6. **Mean Bliss score correlations for vertical inhibition of the KRAS pathway by batoprotafib or BI-3406 in combination with trametinib or temuterkib**. Scatter plots depict significant correlations between the mean Bliss scores from combinations of (A) batoprotafib with trametinib or temuterkib, (B) BI-3406 with trametinib or temuterkib, (C) trametinib with batoprotafib or BI-3406, and (D) temuterkib with batoprotafib or BI-3406. Pearson correlation coefficients (r), confidence intervals (CI), and p-values are shown, with statistical significance indicated by an asterisk (*).
