# Supplementary material for: RAS Pathway Inhibitors Combined with Targeted Agents Are Active in Patient-Derived Spheroids with Oncogenic KRAS Variants from Multiple Cancer Types
Source: Cancer Res Commun. 2025 Oct 8;5(10):1779–95. doi: 10.1158/2767-9764.CRC-24-0582 (PMC12505081; doi:10.1158/2767-9764.CRC-24-0582)
Supplement: Figure S13 — Combination activity of BI-3406 with sapanisertib in multicell-type tumor spheroids. [file crc-24-0582_figure_s13_suppsf13.pdf]

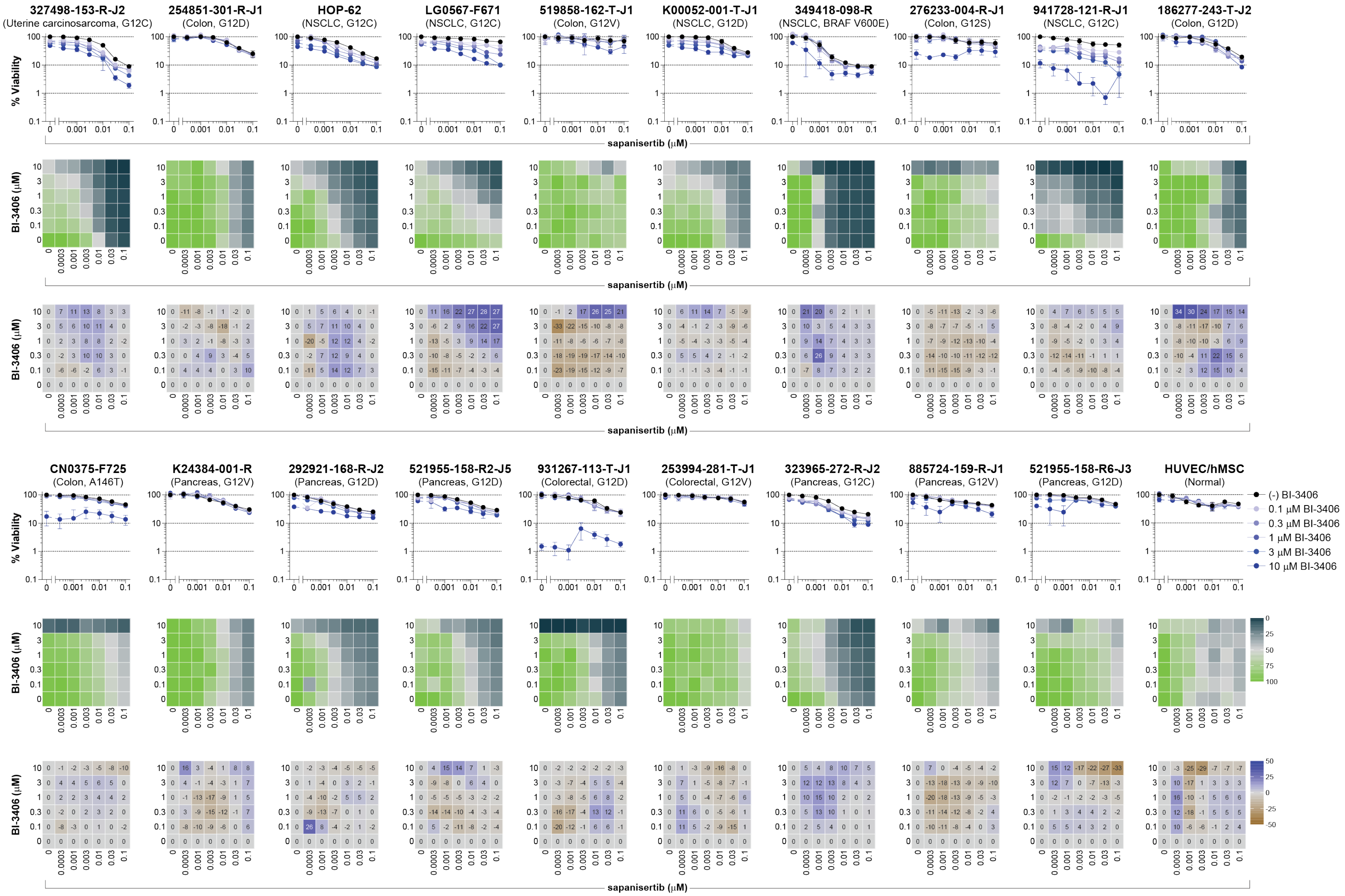

Figure S13. **Combination activity of BI-3406 with sapanisertib in multicell-type tumor spheroids**. Concentration-response graphs (*top*, mean ± SD, n = 4 technical replicates), % viability across the combination's concentration matrix (*middle*, mean of n = 4 technical replicates) displayed as a heatmap (green indicates high cell viability and black indicates low cell viability), and Bliss independence scores across the combination's concentration matrix (*bottom*, mean of n = 4 technical replicates) displayed numerically and as a heatmap (blue indicates synergy, gray indicates additivity, and brown indicates antagonism). Data are shown from nineteen malignant cell lines grown as multicell-type tumor spheroids with HUVEC and hMSC following exposure to BI-3406 and sapanisertib as single agents and in combinations. Data are also shown from HUVEC and hMSC grown as spheroids (mean of n = 3). The malignant cell line name, tumor type, and KRAS status are indicated above each set of graphs.
