# Supplementary material for: RAS Pathway Inhibitors Combined with Targeted Agents Are Active in Patient-Derived Spheroids with Oncogenic KRAS Variants from Multiple Cancer Types
Source: Cancer Res Commun. 2025 Oct 8;5(10):1779–95. doi: 10.1158/2767-9764.CRC-24-0582 (PMC12505081; doi:10.1158/2767-9764.CRC-24-0582)
Supplement: Figure S17 — Sensitivity profiles of the 19 multicell-type tumor spheroids from all RAS pathway inhibitor combinations. [file crc-24-0582_figure_s17_suppsf17.pdf]

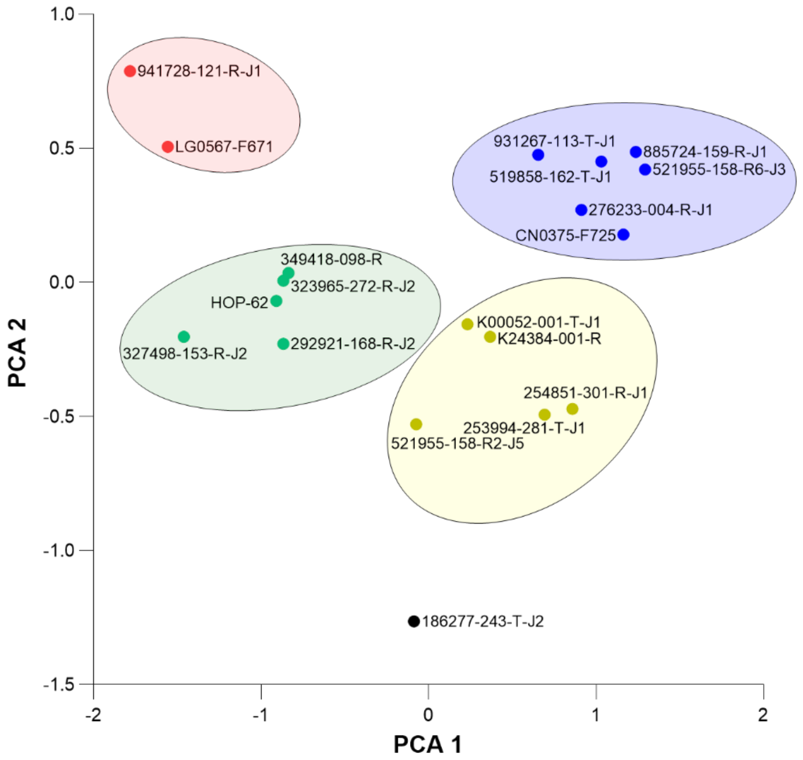

Figure S17. **Sensitivity profiles of the 19 multicell-type tumor spheroids from all RAS pathway inhibitor combinations**. A principal component analysis (PCA) was performed with all nineteen multicell-type tumor spheroid models using two summary metrics of response to all RAS pathway inhibitor combinations. Volume Under the Surface (VUS) is a single metric to describe the % viability data from one multicell-type tumor spheroid model throughout the entire concentration matrix of a drug combination (e.g. lower VUS values indicate greater cytotoxicity across the concentration range). The mean Bliss score, calculated for each spheroid model from an entire drug combination matrix, describes the overall degree and direction of pharmacologic interaction (positive values indicate synergy, negative values indicate antagonism, and values near zero indicate additive responses). Thus, this PCA analysis reveals distinct clusters of tumor spheroid models with similar response profiles based on both cell viability and drug combination effects.
