# Supplementary material for: RAS Pathway Inhibitors Combined with Targeted Agents Are Active in Patient-Derived Spheroids with Oncogenic KRAS Variants from Multiple Cancer Types
Source: Cancer Res Commun. 2025 Oct 8;5(10):1779–95. doi: 10.1158/2767-9764.CRC-24-0582 (PMC12505081; doi:10.1158/2767-9764.CRC-24-0582)
Supplement: Table S1 — Drugs and investigational agents used in this study. The agents included RAS pathway inhibitors (top) and molecular targeted combination drugs (bottom). If available at the time of this study, the clinical Cmax is listed. [file crc-24-0582_table_s1_suppst1.pdf]

Table S1. Drugs and investigational agents used in this study. The agents included RAS pathway inhibitors (*top*) and molecular targeted combination drugs (*bottom*). If available at the time of this study, the clinical C_{max} is listed.

RAS Pathway Inhibitors	Molecular Target	Clinical C_{max}	Dose	Route of Administration	Reference
sotorasib (AMG 510)	KRAS G12C	9.61 µM	960 mg	PO	Nakajima EC, *et. al.* FDA Approval Summary: Sotorasib for KRAS G12C-Mutated Metastatic NSCLC. Clin Cancer Res. 2022 28(8):1482-1486.
batoprotafib (TNO155)	SHP2	*NA*	-	-	-
BI-3406	SOS1	*NA*	-	-	-
**Combination Drugs**	**Molecular Target**	**Clinical C_{max}**	**Dose**	**Route of Administration**	**Reference**
elimusertib (BAY 1895344)	ATR	*NA*	-	-	-
molibresib (GSK525762)	BET bromodomain	*NA*	-	-	-
temuterkib (LY3214996)	ERK	*NA*	-	-	-
venetoclax	BCL-2	4.48 µM	400 mg	PO	Liston DR, Davis M. Clinically Relevant Concentrations of Anticancer Drugs: A Guide for Nonclinical Studies. Clin Cancer Res. 2017 23(14):3489-3498.
alisertib	Aurora A kinase	*NA*	-	-	-
olaparib	PARP	13.1 µM	400 mg	PO	Liston DR, Davis M. Clinically Relevant Concentrations of Anticancer Drugs: A Guide for Nonclinical Studies. Clin Cancer Res. 2017 23(14):3489-3498.
talazoparib	PARP	0.043 µM	1 mg	PO	https://www.accessdata.fda.gov/drugsatfda_docs/label/2018/211651s000lbl.pdf
erdafitinib	FGFR	3.1 µM	8 mg	PO	https://www.accessdata.fda.gov/drugsatfda_docs/label/2019/212018s000lbl.pdf
ipatasertib	AKT1/2/3	*NA*	-	-	-
cabozantinib	cMET, VEGFR, cKIT	4.61 µM	140 mg	PO	Liston DR, Davis M. Clinically Relevant Concentrations of Anticancer Drugs: A Guide for Nonclinical Studies. Clin Cancer Res. 2017 23(14):3489-3498.
nintedanib	PDGFR, FGFR, VEGFR	*NA*	-	-	-
abemaciclib	CDK 4/6	0.588 µM	200 mg	PO	Patnaik A, *et al.* Efficacy and Safety of Abemaciclib, an Inhibitor of CDK4 and CDK6, for Patients with Breast Cancer, Non–Small Cell Lung Cancer, and Other Solid Tumors. Cancer Discovery. 2016 6(7):740-753.
docetaxel	Tubulin stabilizer	5.47 µM	100 mg/m^2	IV	Liston DR, Davis M. Clinically Relevant Concentrations of Anticancer Drugs: A Guide for Nonclinical Studies. Clin Cancer Res. 2017 23(14):3489-3498.
trametinib	MEK 1/2	0.021 µM	2 mg	PO	Liston DR, Davis M. Clinically Relevant Concentrations of Anticancer Drugs: A Guide for Nonclinical Studies. Clin Cancer Res. 2017 23(14):3489-3498.
sapanisertib	mTORC1/2	0.08 µM	3 mg	PO	Al-Kali A, *et al.* A phase 2 and pharmacological study of sapanisertib in patients with relapsed and/or refractory acute lymphoblastic leukemia. Cancer Med. 2023 12(23):21229-21239.
batoprotafib (TNO155)	SHP2	*NA*	-	-	-

NA, clinical C_{max} unknown, the highest concentration tested was 10 µM.
