# Supplementary material for: RAS Pathway Inhibitors Combined with Targeted Agents Are Active in Patient-Derived Spheroids with Oncogenic KRAS Variants from Multiple Cancer Types
Source: Cancer Res Commun. 2025 Oct 8;5(10):1779–95. doi: 10.1158/2767-9764.CRC-24-0582 (PMC12505081; doi:10.1158/2767-9764.CRC-24-0582)
Supplement: Table S2 — The malignant cell lines grown as multicell-type tumor spheroids for this study. The names of both patient-derived and established cell lines are listed along with the tumor type they were derived from and the KRAS status. [file crc-24-0582_table_s2_suppst2.pdf]

Table S2. The malignant cell lines grown as multicell-type tumor spheroids for this study. The names of both patient-derived and established cell lines are listed along with the tumor type they were derived from and the KRAS status.

Cell Line	Tumor Type	KRAS Status	KRAS LOH[a]	KRAS CNA[b]
186277-243-T-J2	Colon cancer	G12D	No	1.9
254851-301-R-J1*	Colon cancer	G12D	No	2.8
276233-004-R-J1	Colon cancer	G12S	No	1.7
519858-162-T-J1	Colon cancer	G12V	No	1.6
CN0375-F725	Colon cancer	A146T	No	1.7
931267-113-T-J1	Colorectal cancer	G12D	No	2.0
253994-281-T-J1	Colorectal cancer	G12V	No	2.0
LG0567-F671	Non-small cell lung cancer	G12C	No	2.0
941728-121-R-J1	Non-small cell lung cancer	G12C	Yes	4.6
K00052-001-T-J1	Non-small cell lung cancer	G12D	No	2.4
349418-098-R	Non-small cell lung cancer	WT (BRAF V600E)	-	1.9
HOP-62	Non-small cell lung cancer	G12C	Yes	4.2
K24384-001-R	Pancreatic cancer	G12V	No	3.7
292921-168-R-J2	Pancreatic cancer	G12D	No	2.6
323965-272-R-J2	Pancreatic cancer	G12C	No	2.1
885724-159-R-J1	Pancreatic cancer	G12V	Yes	2.0
521955-158-R2-J5	Pancreatic cancer	G12D	No	3.6
521955-158-R6-J3	Pancreatic cancer	G12D	No	2.7
327498-153-R-J2	Uterine carcinosarcoma	G12C	No	2.9

Direct links to the NCI Patient-Derived Models Repository and/or cBioPortal are embedded in each model name, providing access to detailed genomic and clinical annotation data.

[a]loss of heterozygosity
[b]copy number alteration
*From the OncoKB Gene Panel of the autologous organoid model (*PDC data were not available*).
