# Supplementary material for: RAS Pathway Inhibitors Combined with Targeted Agents Are Active in Patient-Derived Spheroids with Oncogenic KRAS Variants from Multiple Cancer Types
Source: Cancer Res Commun. 2025 Oct 8;5(10):1779–95. doi: 10.1158/2767-9764.CRC-24-0582 (PMC12505081; doi:10.1158/2767-9764.CRC-24-0582)
Supplement: Table S3 — Cell inoculation densities for multicell-type tumor spheroid models per well in 384-well microplates. [file crc-24-0582_table_s3_suppst3.pdf]

Table S3. Cell inoculation densities for multicell-type tumor spheroid models per well in 384-well microplates.

Malignant Cell Line	Malignant Cells per well	HUVEC[a] per well	hMSC[b] per well
186277-243-T-J2	1250	521	313
254851-301-R-J1	2500	1042	625
276233-004-R-J1	2500	1042	625
519858-162-T-J1	2500	1042	625
CN0375-F725	2500	1042	625
931267-113-T-J1	1250	521	313
253994-281-T-J1	1250	521	313
LG0567-F671	2500	1042	625
941728-121-R-J1	625	260	156
K00052-001-T-J1	625	260	156
349418-098-R	313	130	78
HOP-62	313	130	78
K24384-001-R	625	260	156
292921-168-R-J2	625	260	156
323965-272-R-J2	1250	521	313
885724-159-R-J1	2500	1042	625
521955-158-R2-J5	625	260	156
521955-158-R6-J3	1250	521	313
327498-153-R-J2	625	260	156

[a] human umbilical vein endothelial cells

[b] human mesenchymal stem cells
