# Supplementary material for: RAS Pathway Inhibitors Combined with Targeted Agents Are Active in Patient-Derived Spheroids with Oncogenic KRAS Variants from Multiple Cancer Types
Source: Cancer Res Commun. 2025 Oct 8;5(10):1779–95. doi: 10.1158/2767-9764.CRC-24-0582 (PMC12505081; doi:10.1158/2767-9764.CRC-24-0582)
Supplement: Supplementary Materials & Methods [file crc-24-0582_supplementary_materials__methods_suppsmm.pdf]

Supplementary Materials and Methods

Sotorasib treatment in MIA PaCa-2 3D and 2D culture models. The established pancreatic carcinoma cell line MIA PaCa-2 (ATCC cat. CRL-1420, RRID:CVCL_0428) was purchased from the American Type Culture Collection (ATCC, Manassas, VA) and maintained in DMEM (Invitrogen cat. 11965-118) with 10% FBS (Hyclone cat. SH30070.03). MIA PaCa-2 cells were used to assess sotorasib activity in both three-dimensional (3D) spheroid and two-dimensional (2D) monolayer cultures. For 3D experiments, spheroids were established under two conditions: (1) malignant cells only and (2) malignant cells co-cultured with human umbilical vein endothelial cells (HUVEC) and human mesenchymal stem cells (hMSC). In both 3D conditions, spheroids were seeded in 384-well black/clear round-bottom ULA spheroid microplates (Corning Inc., cat. 3830) with 200 MIA PaCa-2 cells per well (50 µL). In the co-culture condition, an additional 83 HUVEC and 50 hMSC were included per well (50 µL). Following inoculation, microplates were transferred to a Cytomat automated incubator (Thermo Fisher Scientific) and maintained at 37 °C, 5% CO_2, and 95% humidity. Spheroids were allowed to grow for three days prior to treatment. Sotorasib was prepared as an 800× stock solution in dimethyl sulfoxide (DMSO, Sigma-Aldrich, St. Louis, MO, cat. D2650) and dispensed in 62.5 nL volumes into the appropriate wells using an I.DOT non-contact dispenser (DISPENDIX), yielding a final 1× concentration. All treatment conditions were tested in technical triplicate. After seven days of treatment, spheroid viability was assessed by adding 20 µL of CellTiter-Glo 3D reagent (Promega, cat. G9683) to each well. Plates were placed on a microplate shaker for 5 minutes and then incubated at room temperature for an additional 25 minutes. Luminescence was measured using a PHERAstar FSX microplate reader (BMG LABTECH). For 2D experiments, 200 MIA PaCa-2 cells were seeded per well (50 µL) in 384-well white flat bottom polystyrene TC-treated microplates (Greiner Bio-One, cat. 781080) and allowed to adhere for one day prior to treatment. Sotorasib was administered under the same dosing protocol as described above. After three days of treatment, viability was measured using CellTiter-Glo 2.0 (Promega, cat. G9243) per the manufacturer's instructions.

HUVEC/hMSC stromal spheroid model. Three-dimensional spheroids composed solely of endothelial and mesenchymal cells were generated by co-culturing 2,500 HUVEC and 2,500 hMSC per well (50 µL) in 384-well black/clear round-bottom ULA spheroid microplates (Corning Inc., cat. 3830). Following inoculation, the plates were transferred to a Cytomat automated incubator (Thermo Fisher Scientific) and maintained at 37 °C, 5% CO_2, and 95% humidity. Stromal spheroids were allowed to grow for three days prior to treatment. All approved and investigational anticancer agents were delivered using an I.DOT non-contact dispenser (DISPENDIX) by adding 62.5 nL of an 800× DMSO stock solution per well to achieve the desired 1× final concentration. All treatments were performed in technical triplicate. After seven days of exposure, spheroid viability was assessed using the CellTiter-Glo 3D assay (Promega, cat. G9683). A volume of 20 µL of reagent was added to each well, followed by 5 minutes of shaking, and 25 minutes of incubation at room temperature. Luminescence was measured using a PHERAstar FSX microplate reader (BMG LABTECH).
